# Multifaceted atlases of the human brain in its infancy

**DOI:** 10.1038/s41592-022-01703-z

**Published:** 2022-12-30

**Authors:** Sahar Ahmad, Ye Wu, Zhengwang Wu, Kim-Han Thung, Siyuan Liu, Weili Lin, Gang Li, Li Wang, Pew-Thian Yap

**Affiliations:** Department of Radiology and Biomedical Research Imaging Center, University of North Carolina, Chapel Hill, NC USA

**Keywords:** Computational neuroscience, Magnetic resonance imaging, Image processing

## Abstract

Brain atlases are spatial references for integrating, processing, and analyzing brain features gathered from different individuals, sources, and scales. Here we introduce a collection of joint surface–volume atlases that chart postnatal development of the human brain in a spatiotemporally dense manner from two weeks to two years of age. Our month-specific atlases chart normative patterns and capture key traits of early brain development and are therefore conducive to identifying aberrations from normal developmental trajectories. These atlases will enhance our understanding of early structural and functional development by facilitating the mapping of diverse features of the infant brain to a common reference frame for precise multifaceted quantification of cortical and subcortical changes.

## Abstract (web summary)

This Resource presents surface and volume atlases of human brain development during early infancy, at monthly intervals.

## Main

Human brain development is a complex and protracted process that begins during the third gestational week and extends through adulthood^1^. Throughout the late prenatal period and early infancy, the human brain undergoes a myriad of cellular processes, including neurogenesis, neuronal migration, astrogliogenesis, oligodendrogenesis, synaptogenesis, myelination, and synaptic pruning^2^ (Supplementary Fig. 1). These cellular events drive the rapid growth of the infant brain during the first two years of life, resulting in structural changes and reorganization of neural circuits^3^. The intracranial volume of the newborn brain doubles during the first postnatal year, attaining approximately 65% of the adult brain volume^4^. The gray matter (GM) volume increases more (~71%) than the white matter (WM) volume (~20%) in the first year. A precise quantification of early brain growth will be conducive to a step change in our understanding of developments that lead to maturation of cognitive functions^3^. However, endeavors in this direction have so far been hindered by the dearth of brain atlases for mapping features of early brain development to common spaces necessary for fine-grained spatiotemporal quantification of brain changes.

Unlike the long-established growth charts utilized by pediatricians to quantify year-to-year maturation in terms of a child’s height, weight, and head circumference in relation to standardized curves derived from healthy growing children^5^, normative references for neurodevelopment are virtually nonexistent. Recent efforts have been dedicated toward constructing brain charts that capture normative patterns of cerebral development^4^ in terms of volumetric growth of GM, WM, and cerebrospinal fluid (CSF), and cortical growth captured by surface area and cortical thickness^6^. Although these brain charts standardize brain morphological measurements, they do not define a common coordinate system for mapping brain features, particularly from multiple imaging modalities, to a unified reference space. An additional limitation of existing brain charts is that they rely on the demarcation of the brain into adjoint but separate brain areas with hypothetically uniform functions, while in reality an extensive body of evidence suggests a more gradual transition of areal boundaries, particularly in association cortices^7^. These limitations call for the construction of atlases that offer standardized coordinate frameworks for concurrent analysis of multimodal data at multiple levels of granularity.

Existing atlases of the baby brain are typically limited to specific prenatal or postnatal periods^8–12^ (Supplementary Fig. 2 and Supplementary Table 1). Brain atlases densely covering the entirety of the first two years of life are lacking owing to challenges in collecting longitudinal brain magnetic resonance imaging (MRI) data. Adding to the difficulties in constructing these much-needed atlases are the rapid changes in the sizes and shapes of cerebral structures (Supplementary Fig. 3), and the evolving and often insufficient tissue contrast between WM and GM^13^. Moreover, existing cortical surface and volumetric atlases are typically constructed independently, resulting in the following problems:

(i) misalignment between tissue interfaces of the volumetric atlas and the white and pial surfaces of the cortical atlas;
(ii) fuzzy cortical structures, as volumetric displacements are estimated without taking into account the complex convolutions of the cortical surface;
(iii) anatomically implausible displacements owing to cortical surface registration that is based only on surface attributes but ignoring the volumetric data^14^;
(iv) volumetric and cortical surface attributes are not defined in the same space, making it difficult to analyze the two entities concurrently and consistently^15^; and
(v) diminishing of real but subtle changes owing to inconsistent and inaccurate alignment^16^.

In this work, we present a set of month-specific surface–volume longitudinal brain atlases of infants from 2 weeks to 24 months of age. These atlases facilitate the precise mapping of fine-grained changes in both space and time, and are therefore a valuable resource for studying postnatal human brain development, identifying early roots of neurological disorders, and quantifying development-related malformations. We demonstrate that our high-fidelity atlases capture the normative evolutionary landscape of cortical surface features and tissue volumetric characteristics of the infant brain during the first two years of brain development.

## Results

### Surface–volume consistency

Our atlases allow multifaceted analyses of volumetric and surface data in a common space. As a reference, we first construct the infant brain atlas (IBA) at 12 months, for volume (IBA12-V) and surface (IBA12-S), in a surface–volume consistent manner^17^ using high-quality magnetic resonance (MR) images of 37 infant subjects scanned around 12 months of age as part of the Baby Connectome Project (BCP)^18^. The cortical surface and volumetric data of these subjects are simultaneously normalized in space^19^ and then fused to form

(i) the white and pial cortical surfaces of IBA12-S and
(ii) the T1-weighted (T1w) intensity image, T2-weighted (T2w) intensity image, and the WM, GM, and CSF tissue maps of IBA12-V.

**Fig. 1:**
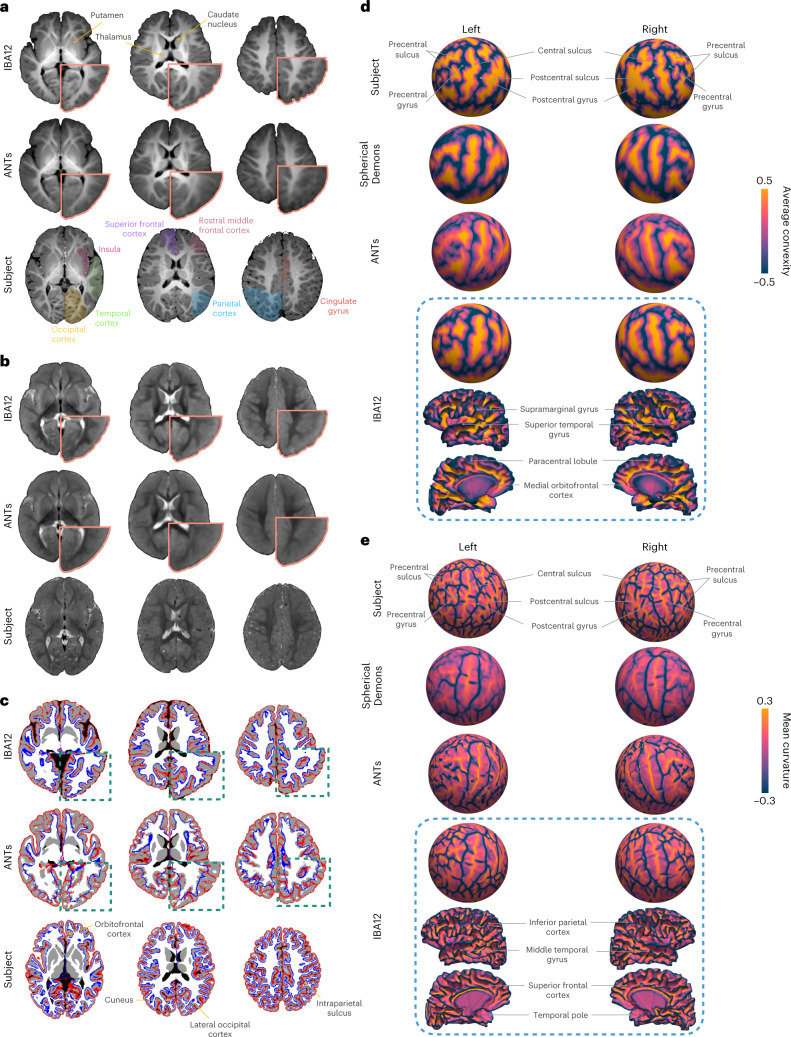
The reference infant brain atlas at **12** months. **a**,**b**, Transverse sections of T1w and T2w atlases and the T1w and T2w images of an individual subject. **c**, White and pial cortical surface atlases overlaid onto the corresponding tissue segmentation atlases. The cortical surfaces of the individual subject are overlaid onto its tissue segmentation map. **d**, Subject and atlas average convexity maps projected onto a sphere. Lateral and medial views of the IBA12-S white surface colored by average convexity are shown for reference. **e**, Subject and atlas mean curvature maps projected onto a sphere. Lateral and medial views of the IBA12-S white surface colored by mean curvature are shown for reference.

In contrast to atlases constructed with Spherical Demons^20^ and ANTs^21^, IBA12-V, and IBA12-S exhibit sharp anatomical details despite being an average of a few tens of subjects (Fig. 1), thanks to the alignment of GM–WM boundaries via explicit registration of cortical surfaces. In fact, IBA12-V resembles MR images of one-year-olds with substantially less fuzziness than the atlas constructed with ANTs without leveraging the geometry of cortical surfaces. This is evident from the close-ups of the temporal and parietal lobes (Fig. 1a,b). Subcortical structures, including the thalamus, caudate nucleus, and putamen, are well defined, indicating that the surface–volume consistent atlas construction process is conducive to preserving both cortical and subcortical anatomical details. While the T1w IBA12-V exhibits adult-like contrast, the T2w IBA12-V is close to isointense with substantial overlap in intensity distributions between WM and GM, consistent with T2w images of one-year-olds^22^. This is particularly noticeable at the superior frontal and rostral middle frontal cortices (Fig. 1b).

The white and pial cortical surfaces of IBA12-S are consistently aligned with the GM–WM and WM–CSF interfaces as delineated by the tissue segmentation maps of IBA12-V (Fig. 1c). This is confirmed by the zoomed-in views of, for example, the lateral occipital, inferior parietal, cuneus, precuneus, medial orbitofrontal, and superior parietal cortices. On the other hand, the ANTs atlas shows inconsistent alignment of the cortical surfaces with tissue interfaces in the volumetric space.

The cortical surfaces of both hemispheres in IBA12-S preserve cortical convolutions with distinct gyral and sulcal patterns (Fig. 1d,e). For greater clarity, we map the average convexity and mean curvature of the white surface onto a sphere. Mapping is only performed for IBA12-S and ANTs as Spherical Demons outputs only spherical atlases of cortical features. Average convexity captures coarse-scale geometric features of primary sulcal folds^23^, whereas mean curvature captures fine-scale geometric features of secondary and tertiary folds. The average convexity maps of the Spherical Demons and IBA12-S atlases are consistent with that of an individual subject. On the other hand, the average convexity map of the ANTs atlas indicates atypical narrowing of the gyral and sulcal folds. Only IBA12-S exhibits typical mean curvature patterns. Spherical Demons gives a fuzzy mean curvature map that fails to capture fine-grained details of cortical folds. ANTs gives an atypical mean curvature map, indicating alteration of surface topology. These results indicate that primary gyri and sulci, including the precentral and postcentral gyri and sulci and the central sulcus, are captured by all three atlases, but localized gyral and sulcal details are only preserved in IBA12-S.

### Surface atlases across infancy

**Fig. 2:**
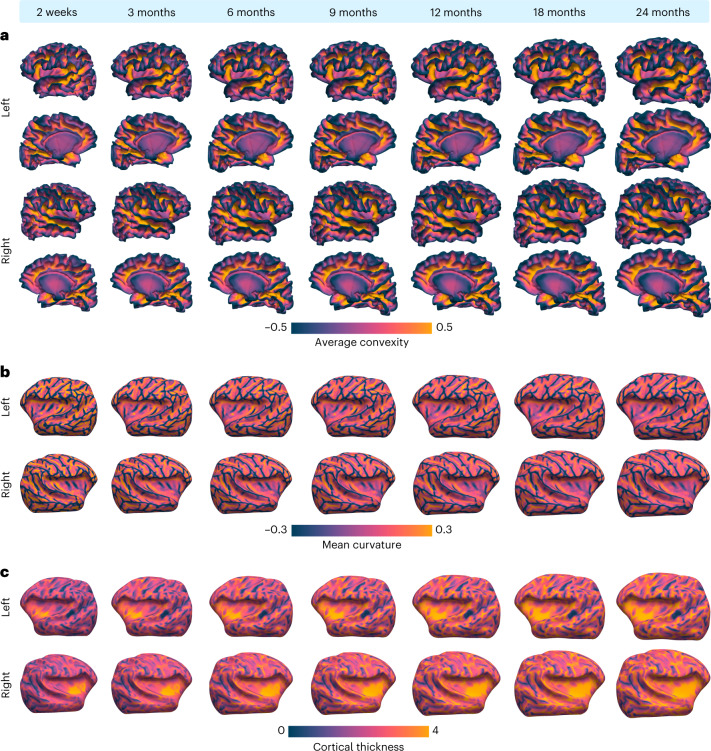
Longitudinal white surface atlases of the infant brain. **a**, Lateral and medial views of white surface atlases from 2 weeks to 24 months, colored by average convexity (mm). **b**, Inflated white surface atlases colored by mean curvature (mm^−1^). **c**, Inflated white surface atlases colored by cortical thickness (mm).

**Extended Data Fig. 1:**
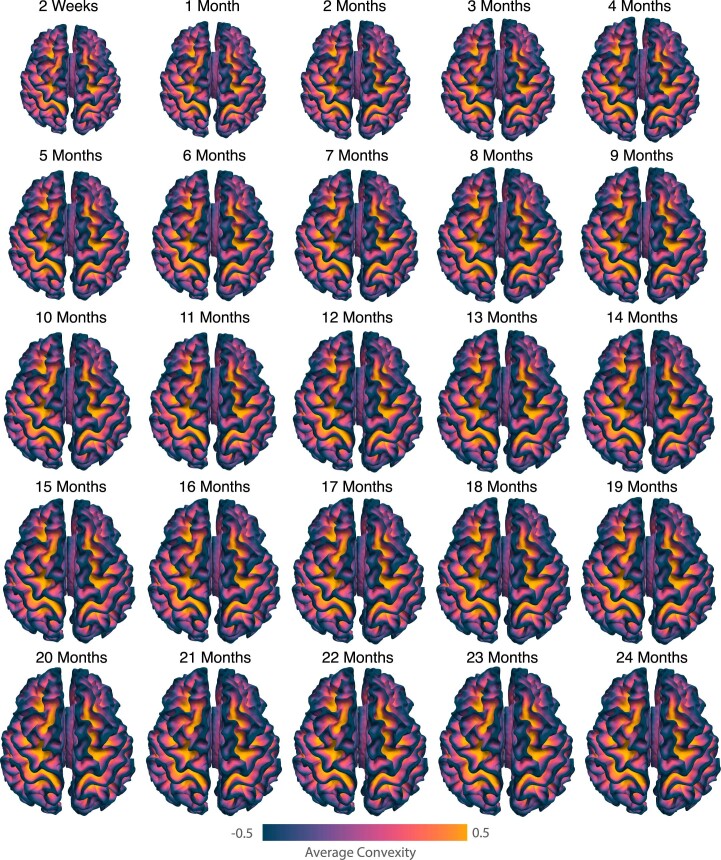
Cortical atlases of the white surface. The age-specific cortical atlases of the white surface for both hemispheres, colored by average convexity (millimeter).

**Extended Data Fig. 2:**
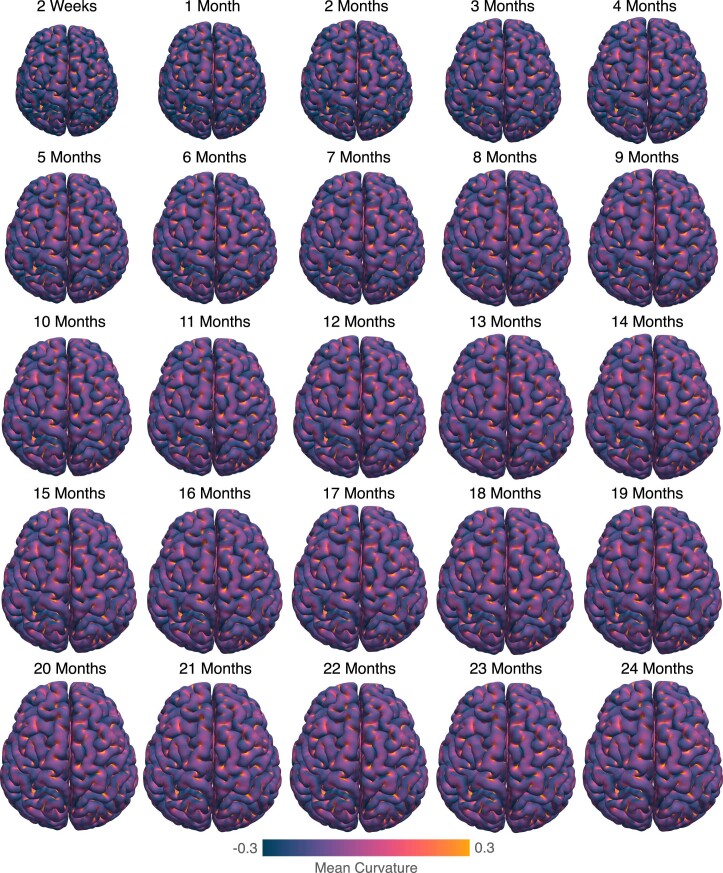
Cortical atlases of the pial surface. Dorsal views of the age-specific cortical atlases of the pial surface for both hemispheres spanning 2 weeks to 24 months, colored by mean curvature (millimeter^−1^).

**Extended Data Fig. 3:**
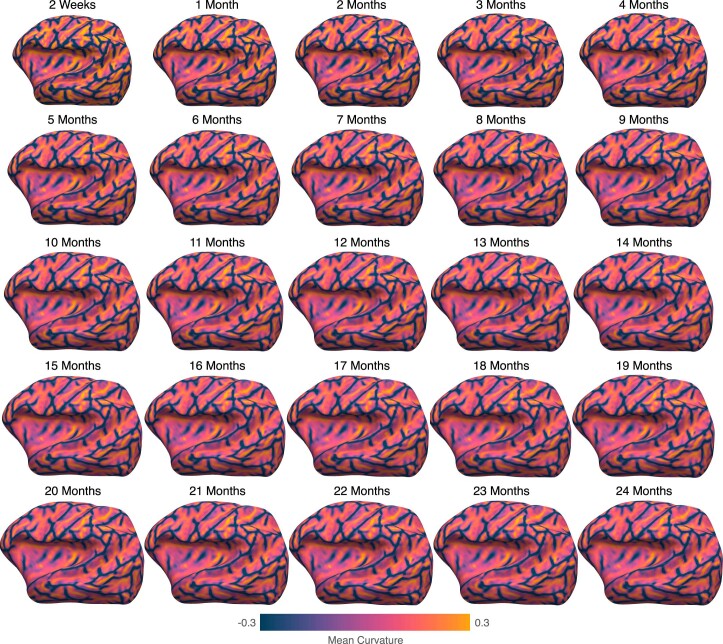
Mean curvature maps of the longitudinal infant brain atlases. The inflated cortical atlases of the white surface (left hemisphere, lateral view) superimposed with mean curvature (millimeter^−1^) spanning 2 weeks to 24 months.

Early brain development is characterized by dynamic changes in cortical folding patterns. We constructed month-specific cortical atlases for the first two years of life by longitudinally warping IBA12-S. Major cortical folds of white surfaces—including the central sulcus, precentral and postcentral gyri, inferior parietal lobule, temporal gyrus, superior and inferior temporal sulci, superior frontal gyrus, cingulate gyrus and sulcus, and parieto-occipital sulcus—are consistent across time points (Fig. 2a and Extended Data Figs. 1–3). Mean curvature mapped onto the inflated white surface atlases indicates subtle developments of secondary and tertiary gyri and sulci (Fig. 2b). Cortical thickness measured between the white and pial cortical surfaces of the atlases, projected onto the inflated white surface atlases (Fig. 2c), indicates that the cortical thickness of the prefrontal cortex, temporal lobe, and insula increases. By contrast, the thickness of the visual and sensorimotor cortices increases at a slower pace.

### Volumetric atlases across infancy

**Fig. 3:**
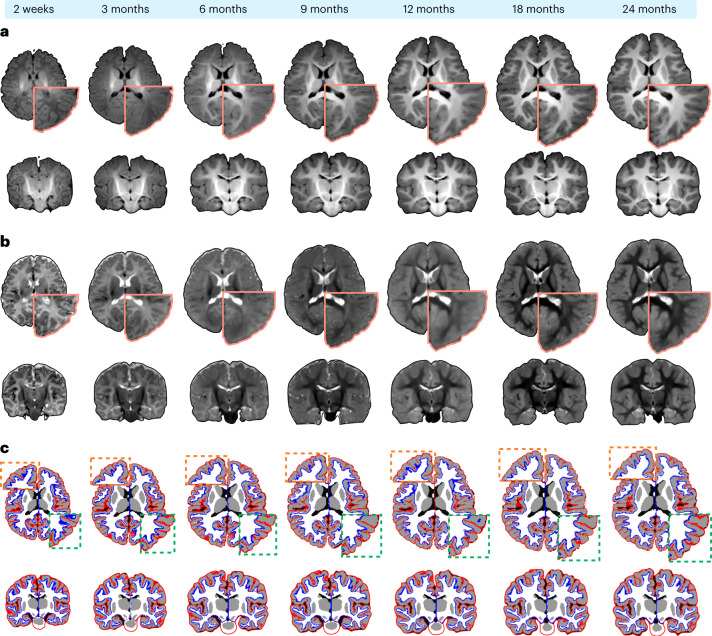
Volumetric atlases of the infant brain. **a**,**b**, Transverse and coronal sections of the T1w and T2w atlases from 2 weeks to 24 months. **c**, White (*blue*) and pial (*red*) surface atlases overlaid onto the corresponding tissue segmentation atlases.

**Extended Data Fig. 4:**
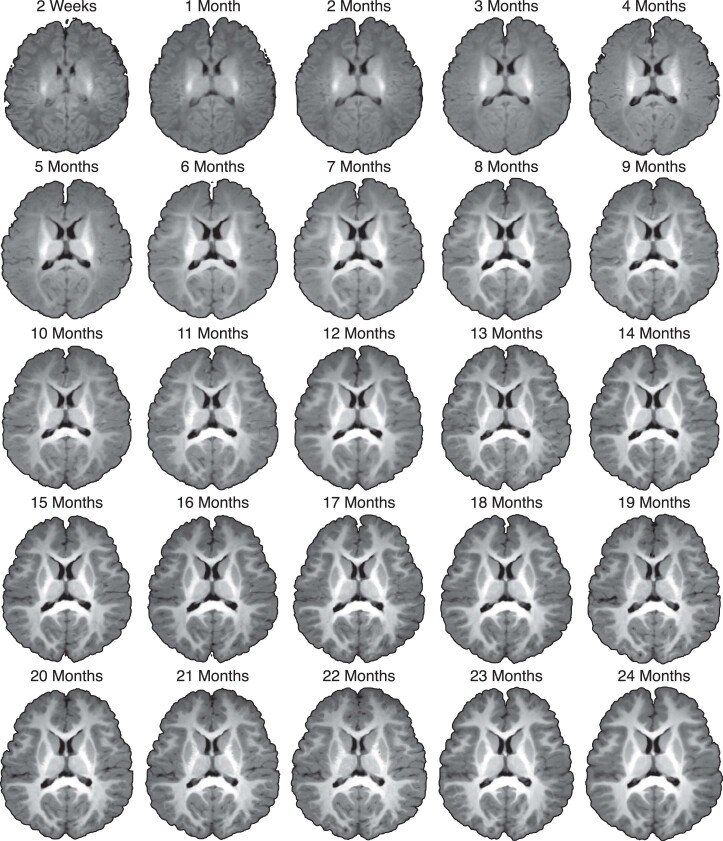
T1w atlases of the infant brain. Transverse sections of the T1w atlases depicting dynamic changes in tissues contrast, size, and shape of anatomical structures at each month between 2 weeks and 24 months.

**Extended Data Fig. 5:**
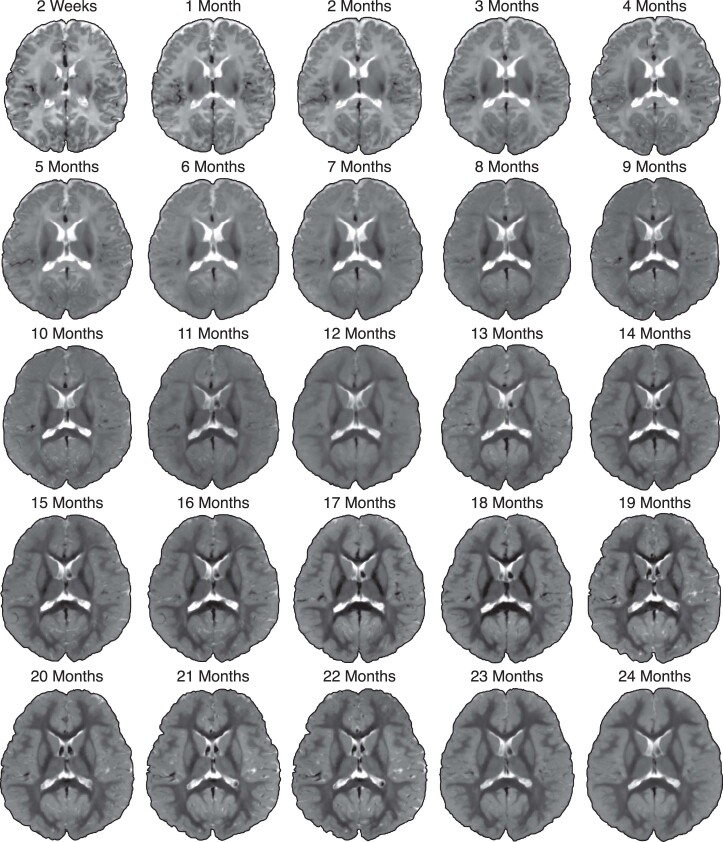
T2w atlases of the infant brain. Transverse sections of the T2w atlases depicting dynamic changes in tissues contrast, size, and shape of anatomical structures at each month between 2 weeks and 24 months.

We generated IBA-V, T1w and T2w atlases from infants 2 weeks to 24 months of age (Fig. 3a,b and Extended Data Figs. 4 and 5). The image contrast evolves month-to-month during year one and becomes adult-like in year two. The T1w and T2w atlases are isointense around 3–6 months and 9–12 months, respectively. The atlases preserve distinct boundaries at the GM–WM interface as evident from the close-ups of the precuneus, cuneus, and inferior parietal cortex (Fig. 3a,b).

**Extended Data Fig. 6:**
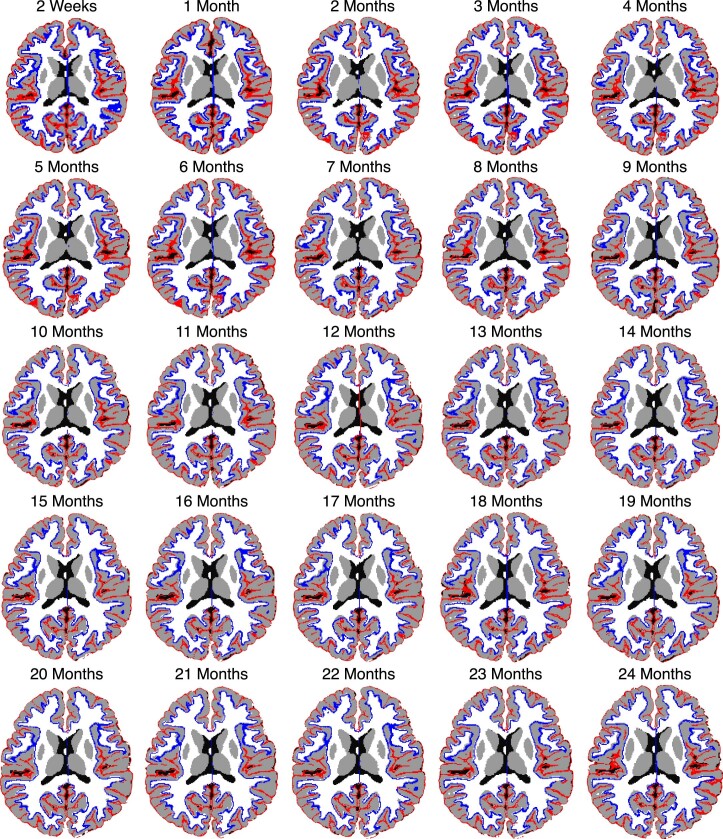
Spatial consistency between age-specific cortical surface and volumetric atlases. Left and right cortical atlases of the white (*blue*) and pial (*red*) surfaces are superimposed onto the tissue segmentation atlases.

Age-specific white and pial cortical surface atlases overlapped on the tissue segmentation atlases (Fig. 3c and Extended Data Fig. 6) indicate that the cortical surface atlases are well aligned with the tissue boundaries of the volumetric atlases, particularly at the superior and middle frontal gyri of both the hemispheres, supramarginal gyrus, inferior parietal cortex, insula, and precentral and postcentral gyri. Good alignment is confirmed by zoomed-in views of the atlases at selected time points (Fig. 3c).

### Surface and volumetric development

**Fig. 4:**
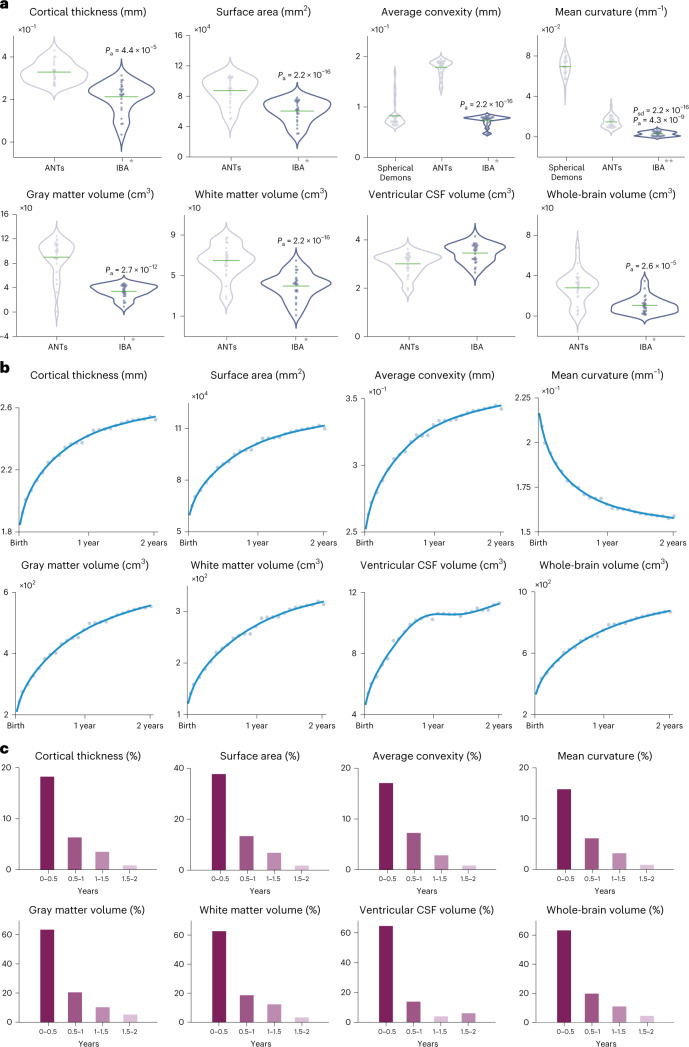
Development of surface and volume. **a**, Violin plots for absolute error of the developmental trajectories of surface and volumetric features, computed between the atlases and the individual subjects. The mean absolute error is marked by a green crossbar. A single star indicates that the mean error for the IBA is significantly different from ANTs (two-tailed paired *t*-test, *P*_a_). A double star indicates that the mean error for the IBA is significantly different from both Spherical Demons (two-tailed paired *t*-test, *P*_sd_) and ANTs (two-tailed paired *t*-test, *P*_a_). **b**, Developmental trajectories (adjusted *R*^2^ = 0.99) of white surface features and tissue volumes of the IBA. **c**, Surface and volumetric changes every six months. Source data

The IBA captures the developmental features (Supplementary Fig. 4) of typically developing infants in the first two years of life. Compared with the atlases constructed with Spherical Demons and ANTs, the IBA more faithfully reflects the individuals, as evidenced by the smaller mean absolute error between the growth curve for each surface or volumetric feature of the IBA and the population curve obtained by fitting the generalized additive mixture model (GAMM)^24^ (Fig. 4a).

We studied the surface and volumetric features captured by the IBA (Fig. 4b,c). During the first and second postnatal years, the IBA-S increases in cortical thickness by 25.7% and 4.3%, increases in surface area by 56.1% and 8.5%, increases in average convexity by 25.5% and 3.6%, and decreases in mean curvature by 20.9% and 3.9% (growth rates are reported in terms of percentage change). During the same period, the IBA-V increases in GM volume by 97.2% and 13.8%, increases in WM volume by 92.9% and 15.9%, increases in ventricular CSF volume by 89.8% and 10.5%, and increases in whole-brain volume (WBV = GM + WM) by 95.6% and 14.6%. We also report the velocity curves along with peak growth ages, indicating maximal growth rates, for these surface and volumetric features (Supplementary Fig. 5).

**Fig. 5:**
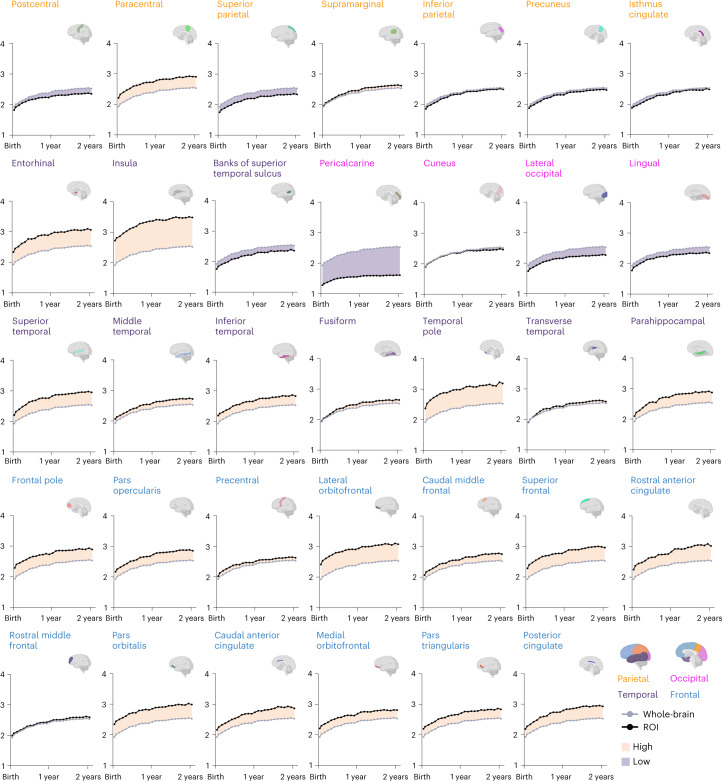
Regional developmental trajectories of cortical thickness. Growth curves of cortical thickness for the IBA cortical regions. Shaded regions indicate whether cortical thickness is higher or lower than the whole-brain average. Source data

**Extended Data Fig. 7:**
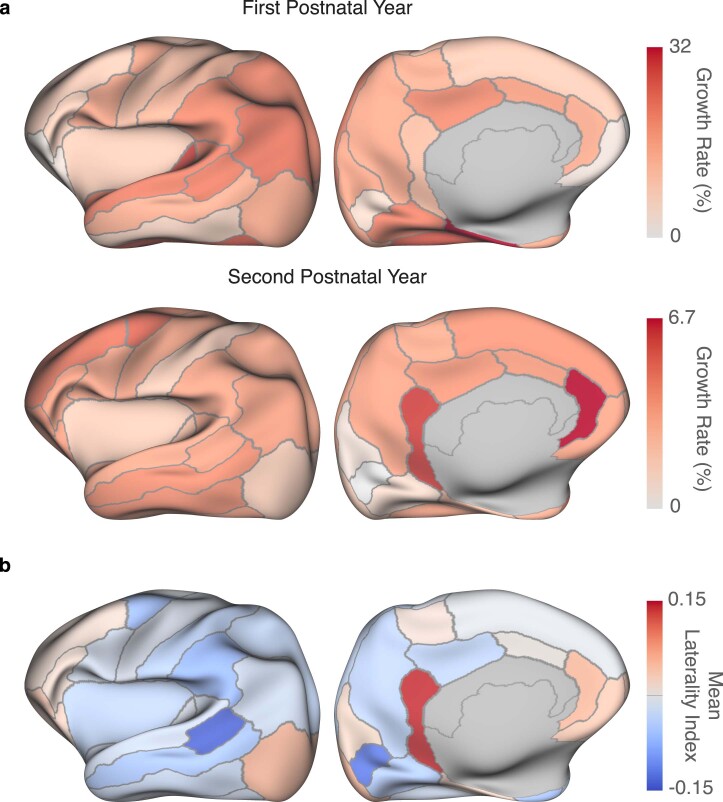
Analysis of cortical thickness. **a**, Regional growth rates in terms of cortical thickness for the first (*top row*) and second (*bottom row*) postnatal years. **b**, ROI-specific mean laterality index for cortical thickness.

Regional analysis indicates spatially heterogeneous development. We measured the thickness of 34 cortical regions-of-interest (ROIs) delineated via FreeSurfer using the Desikan–Killiany atlas^25^ (Supplementary Fig. 6). Developmental trajectories of regional cortical thickness show that the insula, frontal cortex, and superior, middle, and inferior temporal cortices are consistently thicker during infancy (Fig. 5). On the other hand, the precentral and postcentral gyri, occipital and inferior parietal cortices, and banks of superior temporal sulcus are thinner throughout the study period. Regional growth rates vary from 21.2% to 32.0% and 2.3% to 6.7% for the first and second postnatal years (Extended Data Fig. 7). The growth rates (Year 1, Year 2) are (22.6%, 2.3%) for the primary visual cortex, (25.1%, 3.3%) for the primary somatosensory cortex, (24.5%, 4.3%) for the primary motor cortex, (29.5%, 4.0%) for the primary auditory cortex, (27.7%, 4.4%) for the temporal association cortex, (27.6%, 4.4%) for the parietal association cortex, and (23.9%, 4.8%) for the prefrontal association cortex (Extended Data Fig. 7a).

**Extended Data Fig. 8:**
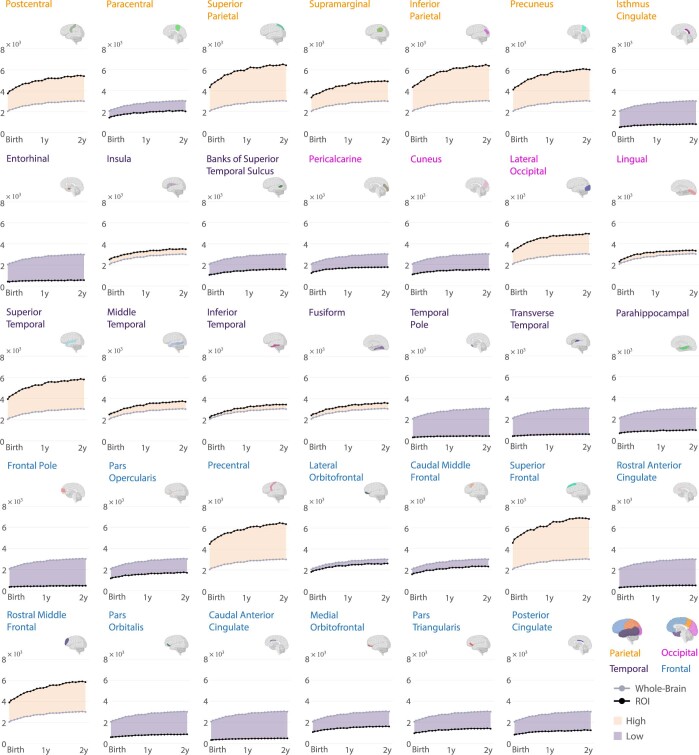
Regional developmental trajectories of surface area. Growth curves of surface area for the IBA cortical regions. Shaded regions indicate whether surface area is higher or lower than the whole-brain average. Source data

**Extended Data Fig. 9:**
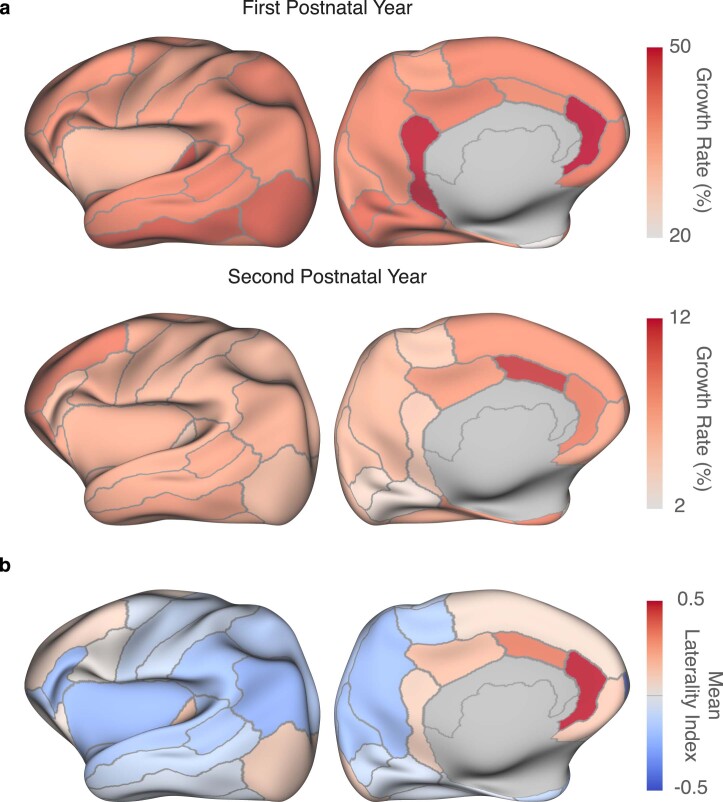
Analysis of surface area. **a**, Regional growth rates in terms of surface area for the first (*top row*) and second (*bottom row*) postnatal years. **b**, ROI-specific mean laterality index for surface area.

We show the developmental trajectories of regional surface area in Extended Data Fig. 8 and growth rates for the first and second postnatal years in Extended Data Fig. 9. High-expansion regions include rostral anterior cingulate gyrus, medial orbitofrontal gyrus, superior frontal gyrus, rostral middle frontal gyrus, inferior and middle temporal gyri, and superior and inferior parietal gyri. Low-expansion regions include insula, pericalcarine, cuneus, entorhinal, temporal pole, and parahippocampal gyrus. Regional growth rates vary from 20.5% to 49.7% in year 1 and 2.9% to 11.1% in year 2. The growth rates (Year 1, Year 2) are (42.4%, 3.4%) for the primary visual cortex, (36.0%, 5.6%) for the primary somatosensory cortex, (34.1%, 6.0%) for the primary motor cortex, (42.3%, 5.3%) for the primary auditory cortex, (39.8%, 6.9%) for the temporal association cortex, (38.9%, 5.8%) for the parietal association cortex, and (38.1%, 7.7%) for the prefrontal association cortex (Extended Data Fig. 9a).

**Extended Data Fig. 10:**
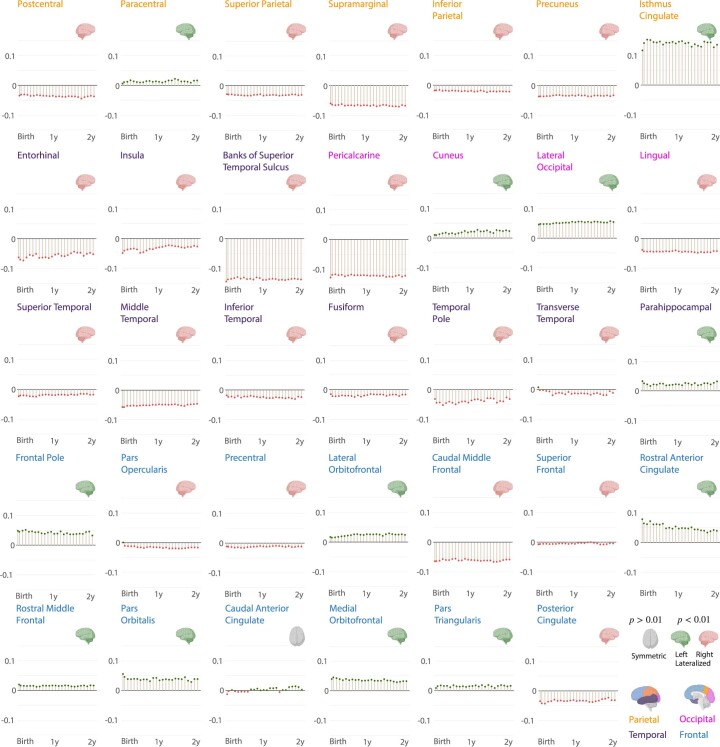
Hemispheric asymmetry of cortical thickness. Region-specific laterality index for cortical thickness of the IBA. Positive laterality is associated with left lateralization (two-tailed *t*-test: p < 0.01) and negative laterality is associated with right lateralization (two-tailed *t*-test: p < 0.01). Source data

The IBA captures the asymmetry of cortical features. We show the hemispheric lateralization of regional cortical features via the laterality index, LI = (left − right)/(left + right), computed for each ROI. There is significant asymmetry (two-tailed *t*-test; *P* < 0.01) in cortical thickness (Extended Data Fig. 10) and surface area (Supplementary Fig. 7) for majority of the ROIs. The corresponding *P* values, *t*-scores, and degrees of freedom are reported for cortical thickness in the source data of Extended Data Fig. 10 and for surface area in Supplementary Data File 2. The ROI-specific mean LI for cortical thickness and surface area are shown in Extended Data Figs. 7b, 9b. Results for other attributes are presented in Supplementary Note 1.

### Cortical T1w/T2w ratio

**Fig. 6:**
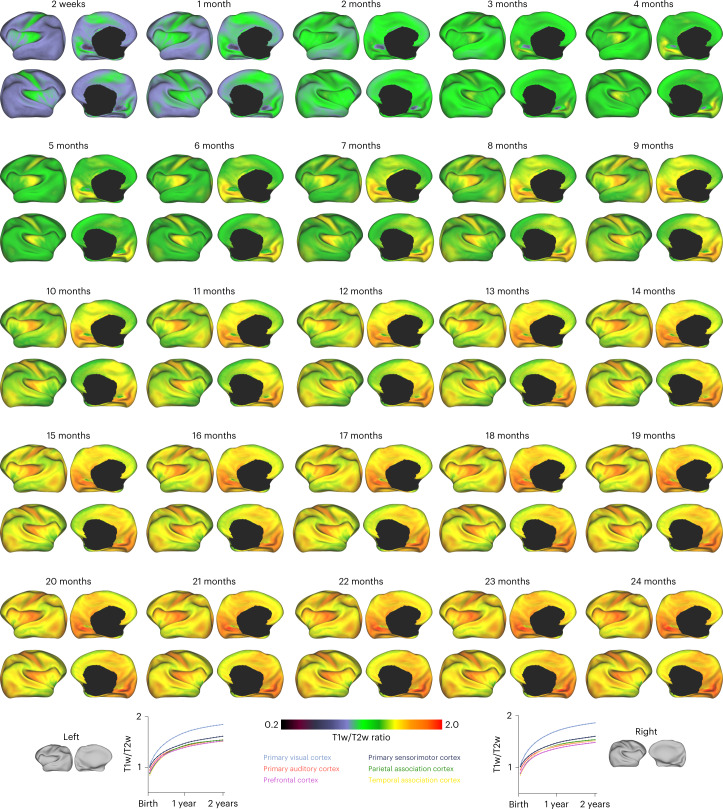
T1w/T2w ratio across infancy. Cortical T1w/T2w ratio, mapped onto the inflated white surfaces of the IBA. Source data

We investigated cortical myelination across infancy by T1w/T2w ratio mapping of the cortical ribbon to the white surface of each individual subject^26^. The GAMM-fitted myelin maps (Fig. 6) indicate increasing myelination in both cerebral hemispheres at average rates of 57.7% and 7.9% in the first and second postnatal years, respectively. Myelination is spatially heterogeneous with heavy myelination in the primary visual, motor, and somatosensory cortices and light myelination in the prefrontal, parietal, and temporal association cortices.

## Discussion

Charting normative structural and functional changes during early brain development is key to understanding aberrations associated with neurodevelopmental disorders such as attention-deficit/hyperactivity disorder, dyslexia, and cerebral palsy^27–29^. We have presented a set of longitudinal, unified surface–volume atlases of the infant brain covering every month of the first two postnatal years. These atlases represent a resource that will facilitate the joint investigation of changes in cortical geometries and brain tissues during a period of rapid and critical brain development. Our atlases are substantially better in preserving anatomical details and surface geometric features than ANTs^21,30^ and Spherical Demons^20,31^.

The human brain undergoes complex gyrification that begins after mid-gestation^32^, nudging the initially smooth cerebral surface into a highly convoluted structure. A widely used macroscopic morphological measure is cortical thickness, which is systematically related to the cytoarchitecture and hierarchical structural organization of the cortex^33^. The neurobiological mechanisms underlying developmental changes in cortical thickness are complex and involve processes such as synaptogenesis, proliferation of dendrites and dendritic spines, axonal sprouting, and vascular development^34^. The IBA shows that cortical thickness increases rapidly during the first year and more slowly in the second year. Greater cortical thickness during early brain development is in general positively associated with intelligence and cognitive skills in later stages of life^35^. The IBA also indicates that cortical thickness changes heterogeneously across the cortex, in line with previous studies^36^. The association cortices in the temporal, parietal, and prefrontal lobes are thicker compared with the primary visual and sensorimotor cortices, which agrees with the fact that thicker cortices are in general less mature. This is consistent with the functional development of the infant brain where vision, motor, and sensory systems are more mature than executive functions. More discussion on the cortical attributes of the IBA is included in Supplementary Note 2.

In addition to folding geometry, we quantitatively assessed brain tissue volume changes during infancy. Early neurodevelopment is underpinned by cellular and molecular processes that drive the growth and maturation of brain tissues. Cell bodies, axon terminal branches, dendrites, and spines residing in GM contribute to the early growth of GM^37^. Axonal tracts accounting for WM form inter-hemispheric, cortico-cortical, limbic, brainstem, and cortico-spinal connections, providing an efficient network of structural connectivity^38,39^. Furthermore, CSF, apart from cushioning the brain, contributes to brain maturation and function by delivering growth factors and signaling molecules to progenitor cells that proliferate into immature neurons, which later migrate to different areas of the cerebral cortex^40,41^. IBA growth curves for GM, WM, ventricular CSF, and whole-brain volume indicate that volumes of all tissues increase from birth through two years of age, albeit at rates different from previous studies. Knickmeyer et al.^4^ reported changes in GM volume by 149% in the first year and 14% in the second year. More moderate changes were reported for the WM volume at 11% in the first year and 19% in the second year. On the other hand, the IBA increases in GM volume by 97.2% and 13.8%, and increase in WM volume by 92.9% and 15.9%, during first and second postnatal years. These discrepancies can be due to differences in segmentation methods, percentage change definitions, and what were considered as WM and GM during volume calculation.

Overall, qualitative and quantitative analyses confirm that the surface–volume consistent IBA faithfully reflects the growth trajectories of infants with rich anatomical details. These atlases capture monthly changes in brain shape and size, cortical geometry, tissue contrast, volume, and microstructural characteristics of typically developing infants. A trait that sets the IBA apart from currently available atlases is that it covers each month during the first two postnatal years, providing a dense spatiotemporal depiction of this critical period of development. We hope that these atlases will become an invaluable common coordinate framework and facilitate the discovery of new insights into developmental processes underpinning child cognition and social behavior.

## Methods

### Dataset and preprocessing

Longitudinal T1w and T2w MRI scans of 37 subjects (20 females and 17 males) enrolled as part of the UNC/UMN Baby Connectome Project (BCP)^18^ were used in this work. Participants were recruited from existing registries at UNC and UMN based on state-wide birth records as well as from broader community resources (for example, community centers and targeted day-care centers) to ensure the sample approximates the racial/ethnic and socio-economic diversity of the US census. Informed consent was obtained from the parents of all subjects. The subjects were divided into six cohorts (A_1_, A_2_, A_3_, B_1_, B_2_, and B_3_) with first visits scheduled at 2 weeks, 1, 2, 9, 10, and 11 months, respectively. The subjects in A_1_, A_2_, and A_3_ were scheduled to be scanned every 3 months in the first year and then at 24 months; whereas, the subjects in B_1_, B_2_, and B_3_ were scanned every 3 months for the first 2 years. The total number of scans for each subject is different since not all subjects could be scanned at all expected time points. A total of 108 scans for each imaging modality were used. The study protocols were approved by the Institutional Review Board of the School of Medicine of The University of North Carolina at Chapel Hill.

The images were acquired using 3T Siemens Prisma MRI scanners equipped with Siemens 32-channel head coils. T1w MR images were acquired with 208 sagittal slices, TR/TE = 2,400/2.24 ms, flip angle = 8°, acquisition matrix = 320 × 320, and resolution = (0.8 mm)^3^. T2w MR images were acquired with 208 sagittal slices, TR/TE = 3,200/564 ms, variable flip angles, acquisition matrix = 320 × 320, and resolution = (0.8 mm)^3^.

MR images were quality-controlled using an automated algorithm^42^ and then preprocessed using an infant-centric processing pipeline (iBEAT v.2.0; available at https://ibeat.wildapricot.org) consisting of the following steps:

(i) rigid alignment of T1w and T2w images using FLIRT^43^;
(ii) skull stripping by a learning-based method^44^;
(iii) intensity inhomogeneity correction by N3^45^;
(iv) brain tissue segmentation by an infant dedicated learning-based method^46^;
(v) hemisphere separation and subcortical filling; and
(vi) topologically-correct cortical surface reconstruction^47^.

Example results of the processing steps are shown in Supplementary Fig. 15.

### Surface–volume atlas construction

Our atlas construction method (Supplementary Fig. 16a) involves two main tasks:

(i) construction of the 12-month surface–volume atlas via surface-constrained groupwise registration; and
(ii) construction of longitudinal atlases for 2 weeks to 24 months by propagating the 12-month atlas through parallel transported longitudinal deformations.

The steps involved in each task are detailed below.

*(i) Construction of reference atlas*—*IBA12.* We first construct the 12-month surface–volume reference atlas, that is, IBA12, and use it to generate the atlases at the other time points. The IBA12 lies in the middle of the two-year time span and is hence ideal to capture developmental patterns between birth and two years of age.

Surface-constrained groupwise registration. The low intensity contrast of infant brain MRI renders image registration and atlas construction challenging. Here we use a dynamic elasticity model with surface constraint (SC-DEM^19^) for groupwise registration using tissue segmentation maps instead of intensity images. Groupwise registration allows a population of tissue segmentation maps to be registered simultaneously to a common space as demonstrated in our previous work^17^. In our case, this is achieved by first selecting a reference based on a subject scan that is most similar in appearance to the whole set of images. Then, the reference tissue segmentation map is iteratively updated by fusing all tissue segmentation maps that are registered to it. SC-DEM improves the alignment of tissue boundaries using predetermined surface transformations. Before SC-DEM groupwise registration, the tissue segmentation maps are affine-aligned with the reference tissue segmentation map using FLIRT^43^. From here on, affine transform is assumed to have taken place before SC-DEM registration unless otherwise stated.

For cortical surface registration, the white surface is inflated and mapped onto the unit sphere via metric distortion minimization^23^. The surfaces in the spherical space are represented by two folding attributes: average convexity and mean curvature. The spherical surfaces of the moving scans are registered to that of the reference scan using Spherical Demons^20^. The resulting vertex-wise correspondences are propagated to the white and pial cortical surfaces by leveraging the one-to-one vertex mapping between the spherical surfaces and the white and pial cortical surfaces.

SC-DEM employs a dynamic elasticity model^48^ to characterize large nonlinear displacements. For the *i*-th subject scanned at time $\tau \in {{{{\mathcal{W}}}}}_{12}$ (in terms of chronological age), where ${{{{\mathcal{W}}}}}_{\rho }=[(\rho -1.5),(\rho +1.5)]$ months, we estimate displacement field with respect to a reference ${\phi }_{(i,\tau )\to {{{\rm{ref}}}}}(\overrightarrow{x})$ by solving the wave equation:

1 $$\begin{array}{l}\frac{{\partial }^{2}{\phi }_{(i,\tau )\to {{{\rm{ref}}}}}(\overrightarrow{x})}{\partial {t}^{2}}=\alpha \left({\nabla }^{2}{\phi }_{(i,\tau )\to {{{\rm{ref}}}}}(\overrightarrow{x})+\nabla \left(\nabla \cdot {\phi }_{(i,\tau )\to {{{\rm{ref}}}}}(\overrightarrow{x})\right)\right)\\+\beta {f}_{(i,\tau )\to {{{\rm{ref}}}}}^{{{{\rm{vol}}}}}(\overrightarrow{x})+\gamma {f}_{(i,\tau )\to {{{\rm{ref}}}}}^{\,{{{\rm{surf}}}}}(\overrightarrow{x}),\end{array}$$

where ∇^2^ is the vector Laplacian operator and ${f}_{(i,\tau )\to {{{\rm{ref}}}}}^{\,{{{\rm{vol}}}}}(\overrightarrow{x})$ and ${f}_{(i,\tau )\to {{{\rm{ref}}}}}^{\,{{{\rm{surf}}}}}(\overrightarrow{x})$ are respectively the volumetric and surface force fields. The volumetric force field ${f}_{(i,\tau )\to {{{\rm{ref}}}}}^{\,{{{\rm{vol}}}}}(\overrightarrow{x})$ captures the misalignment between the warped moving tissue segmentation map ${I}_{(i,\tau )}(\overrightarrow{x}+{\phi }_{(i,\tau )\to {{{\rm{ref}}}}}(\overrightarrow{x}))$ and the reference tissue segmentation map ${I}_{{{{\rm{ref}}}}}(\overrightarrow{x})$:

2 $${f}_{(i,\tau )\to {{{\rm{ref}}}}}^{\,{{{\rm{vol}}}}}(\overrightarrow{x})=\left[{I}_{(i,\tau )}(\overrightarrow{x}+{\phi }_{(i,\tau )\to {{{\rm{ref}}}}}(\overrightarrow{x}))-{I}_{{{{\rm{ref}}}}}(\overrightarrow{x})\right]\frac{\partial {I}_{(i,\tau )}\left(\overrightarrow{x}+{\phi }_{(i,\tau )\to {{{\rm{ref}}}}}(\overrightarrow{x})\right)}{\partial \left(\overrightarrow{x}+{\phi }_{(i,\tau )\to {{{\rm{ref}}}}}(\overrightarrow{x})\right)}.$$

The surface force field ${f}_{(i,\tau )\to {\,{{\rm{ref}}}}}^{{{{\rm{surf}}}}}(\overrightarrow{x})$ is computed on the basis of the differences between the volumetric displacements and the predetermined surface displacements for white and pial surfaces for both left and right hemispheres. The parameter *α* controls deformation smoothness and parameters *β* and *γ* balance the two force fields and control the rate of convergence. Registration halts when the two force fields are negligibly small, thereby consistently aligning the surface and the tissue segmentation map.

Cortical surface atlas. The cortical surface atlas comprising white and pial surfaces of both left and right hemispheres is constructed by using the registered cortical surfaces ${\{{\hat{S}}_{(i,\tau )}\}}_{(i,\tau )}$, each associated with a weight *w*(*τ*, 12), computed using $w({\tau }_{1},{\tau }_{2})=\frac{1}{\sigma \sqrt{2\pi }}\exp \left(\frac{-{({\tau }_{1}-{\tau }_{2})}^{2}}{2{\sigma }^{2}}\right)$, where parameter *σ* controls temporal smoothness and is set to 0.7 months. The 12-month cortical surface atlas ${A}_{12}^{{{{\rm{surf}}}}}$ is constructed by weighted averaging of the vertex coordinates for $\tau \in {{{{\mathcal{W}}}}}_{12}$: $\frac{{\sum }_{(i,\tau )}w(\tau ,12){\hat{S}}_{(i,\tau )}}{{\sum }_{(i,\tau )}w(\tau ,12)}$. Next, we use ${A}_{12}^{{{{\rm{surf}}}}}$ to construct the corresponding volumetric atlas such that the two atlases are defined in the same coordinate space.

Volumetric atlas. The tissue segmentation volumetric atlas ${A}_{12}^{{{{\rm{tissue}}}}}$ is constructed by correcting the displacement fields ${\{{\phi }_{(i,\tau )}(\overrightarrow{x})\}}_{(i,\tau )}$ for $\tau \in {{{{\mathcal{W}}}}}_{12}$ on the basis of the surface atlas ${A}_{12}^{{{{\rm{surf}}}}}$; ensuring the alignment of the volumetric WM–GM and GM–CSF interfaces in accordance with the surface atlas. This involves updating the volumetric displacement fields by considering surface misalignment as described below:

1. Compute for each vertex with coordinates $\overrightarrow{y}$ on the surface atlas the vertex-wise surface displacement ${{\Delta }}{\psi }_{(i,\tau )}(\overrightarrow{y})$ between ${\hat{S}}_{(i,\tau )}$ and ${A}_{12}^{{{{\rm{surf}}}}}$.
2. Find in ${A}_{12}^{{{{\rm{surf}}}}}$ the triangle with vertices ${\{{\overrightarrow{y}}_{p}\}}_{p = 1}^{3}$ closest to a query voxel $\overrightarrow{x}$.
3. Compute the corrective volumetric displacement field ${{\Delta }}{\phi }_{(i,\tau )}(\overrightarrow{x})$ as
  3 $${{\Delta }}{\phi }_{(i,\tau )}(\overrightarrow{x})=\left\{\begin{array}{ll}\frac{\mathop{\sum }\nolimits_{p = 1}^{3}w(\overrightarrow{x},{\overrightarrow{y}}_{p}){{\Delta }}{\psi }_{(i,\tau )}({\overrightarrow{y}}_{p})}{\mathop{\sum }\nolimits_{p = 1}^{3}w(\overrightarrow{x},{\overrightarrow{y}}_{p})}\quad &{{{\rm{if}}}}\ \parallel \overrightarrow{x}-{\overrightarrow{y}}_{p}\parallel \le \delta ,\quad \forall p;\\ 0\quad &{{{\rm{otherwise}}}},\end{array}\right.$$
  where $w(\overrightarrow{x},\overrightarrow{y})=\frac{1}{{\sigma }_{g}\sqrt{2\pi }}\exp \left(\frac{-{\parallel \overrightarrow{x}-\overrightarrow{y}\parallel }^{2}}{2{\sigma }_{g}^{2}}\right)$ and *δ* = 6 mm. We set *σ*_*g*_ = 3 mm for smooth displacement fields.
4. Warp the tissue segmentation maps using the total displacement field ${\phi }_{(i,\tau )\to {A}_{12}^{{{{\rm{vol}}}}}}={\phi }_{(i,\tau )\to {{{\rm{ref}}}}}+{{\Delta }}{\phi }_{(i,\tau )}$ for better alignment and preservation of anatomical details.
5. Determine the tissue label via majority voting for $\tau \in {{{{\mathcal{W}}}}}_{12}$.
6. Warp the intensity images (T1w and T2w) using the total displacement field ${\phi }_{(i,\tau )\to {A}_{12}^{{{{\rm{vol}}}}}}$ to obtain ${\{{\hat{I}}_{(i,\tau )}\}}_{(i,\tau )}$.
7. Average the warped intensity images using the weights {*w*(*τ*,12)} to obtain 12-month T1w and T2w atlases: ${A}_{12}^{{{{\rm{int}}}}}=\frac{{\sum }_{(i,\tau )}w(\tau ,12){\hat{I}}_{(i,\tau )}}{{\sum }_{(i,\tau )}w(\tau ,12)}$.

*(ii) Construction of longitudinal surface–volume atlases.* The surface–volume atlases for 2 weeks to 24 months are generated by propagating the IBA12 to each month. To achieve this, we determine the average longitudinal change from each month to the 12-month atlas space. This is realized by transporting the longitudinal deformations to the atlas space via inter-subject deformations (Supplementary Fig. 16b).

Parallel transport of longitudinal deformations. The longitudinal growth of a subject is characterized by the displacement field ${\phi }_{(i,\tau ^{\prime} )\to (i,\tau )}$ from time point $\tau ^{\prime}$ to time point $\tau \in {{{{\mathcal{W}}}}}_{12}$, estimated via affine and SC-DEM registration. Intra-subject deformation fields ${{{\Phi }}}_{(i,\tau ^{\prime} )\to (i,\tau )}=\overrightarrow{x}+{\phi }_{(i,\tau ^{\prime} )\to (i,\tau )}$ are spatially normalized to the 12-month atlas space by parallel transport^49^—disentangling inter-subject variability from longitudinal growth. The transported deformation field from the subject space to the 12-month atlas space is given as ${{{\Phi }}}_{(i,\tau ^{\prime} )\to (i,\tau )\to {A}_{12}^{{{{\rm{vol}}}}}}^{\parallel }={{{\Phi }}}_{(i,\tau )\to {A}_{12}^{{{{\rm{vol}}}}}}^{-1}\circ {{{\Phi }}}_{(i,\tau ^{\prime} )\to (i,\tau )}\circ {{{\Phi }}}_{(i,\tau )\to {A}_{12}^{{{{\rm{vol}}}}}}$.

Kernel regression. To construct age-specifc surface–volume consistent atlases, we warp the 12-month atlas to each specific time point *τ*_0_ with the weighted average of the transported displacement fields ${\{{\phi }_{(i,\tau ^{\prime} )\to (i,\tau )\to {A}_{12}^{{{{\rm{vol}}}}}}^{\parallel }\}}\,_{\tau ^{\prime} \in {{{{\mathcal{W}}}}}_{{\tau }_{0}},\tau \in {{{{\mathcal{W}}}}}_{12}}$:

4 $${\phi }_{{\tau }_{0}\to {A}_{12}^{{{{\rm{vol}}}}}}^{\parallel }=\frac{{\sum }_{i}w(\tau ^{\prime} ,{\tau }_{0})w(\tau ,12)\ {\phi }_{(i,\tau ^{\prime} )\to (i,\tau )\to {A}_{12}^{{{{\rm{vol}}}}}}^{\parallel }}{{\sum }_{i}w(\tau ^{\prime} ,{\tau }_{0})w(\tau ,12)}.$$

The transported displacement fields are weighted based on whether $\tau ^{\prime}$ and *τ* are close to *τ*_0_ and 12, respectively (Supplementary Fig. 16c). The displacement field ${\phi }_{{\tau }_{0}\to {A}_{12}^{{{{\rm{vol}}}}}}^{\parallel }$ is used to warp the 12-month cortical surface and tissue segmentation maps atlases to each time point. Intensity images (T1w and T2w) are warped with total deformation fields ${{{\Phi }}}_{(i,\tau ^{\prime} )\to (i,\tau )}\circ {{{\Phi }}}_{(i,\tau )\to {A}_{12}^{{{{\rm{vol}}}}}}\circ {{{{\Phi }}}^{\parallel }}_{{\tau }_{0}\to {A}_{12}^{{{{\rm{vol}}}}}}^{-1}$ to obtain ${\{{\hat{I}}_{(i,\tau ^{\prime} )}\}}_{(i,\tau ^{\prime} )}$, which are averaged to generate T1w and T2w atlases: ${A}_{{\tau }_{0}}^{{{{\rm{int}}}}}=\frac{{\sum }_{i}w(\tau ^{\prime} ,{\tau }_{0})w(\tau ,12)\ {\hat{I}}_{(i,\tau ^{\prime} )}}{{\sum }_{i}w(\tau ^{\prime} ,{\tau }_{0})w(\tau ,12)}$.

### Developmental trajectories of the infant population

To characterize infant brain maturation, we estimated the developmental trajectories of brain tissues volume, cortical thickness, surface area, average convexity, mean curvature, and cortical T1w/T2w ratio. For subject *i* at scan time *t*, the age effects on feature *f*_*i*_(*t*) are modeled using GAMM^24^: *f*_*i*_(*t*) = *s*(*t*) + *γ*_*i*_. Here, *s*(⋅) is a smooth nonlinear function represented by cubic regression splines and *γ*_*i*_ is the subject-specific random intercept. The degree of smoothness is selected using the restricted maximum likelihood criterion in R (https://www.r-project.org/).

### Developmental trajectories of the IBA

To characterize developmental pattern captured by the IBA, we estimated the growth trajectories of surface and volumetric features. For the IBA at time point *t*, the age effects on feature *f*(*t*) are modeled using the generalized additive model (GAM): *f*(*t*) = *s*(*t*). Here, *s*(⋅) is a smooth nonlinear function represented by cubic regression splines. The degree of smoothness is selected using the restricted maximum likelihood criterion in R.

### Statistical analysis for laterality

For each cortical ROI, we performed a one-sample two-tailed *t*-test at 1% significance level to determine whether the surface geometric features are significantly lateralized across the first two years of life.

### Atlas construction with compared methods

For comparison purposes, we constructed (i) cortical surface atlases with Spherical Demons^20^ and (ii) volume and surface atlases with ANTs^21^. We used developer-optimized parameters^20,50^.

*(i) Spherical Demons atlas construction.* The cortical surface atlases at each month *τ*_0_ are generated by groupwise registration of white surfaces of the subjects scanned at time $\tau ^{\prime} \in {{{{\mathcal{W}}}}}_{{\tau }_{0}}$. The white cortical surfaces are mapped onto the unit sphere and then spatially aligned via Spherical Demons, which uses average convexity and mean curvature to drive surface registration. During groupwise registration, individual cortical surfaces are aligned to the iteratively updated mean cortical surface. Finally, average convexity and mean curvature maps are averaged to obtain the surface atlases.

*(ii) ANTs atlas construction.* We first generate the 12-month surface and volume atlases, and use these to obtain atlases at the other time points. For the 12-month atlas, the tissue segmentation maps of the subjects scanned at time $\tau \in {{{{\mathcal{W}}}}}_{12}$ are spatially aligned via groupwise registration with ANTs. The cortical surfaces and intensity images of the registered subjects are then averaged with weights {*w*(*τ*, 12)}. The warped tissue segmentation maps are fused via majority voting. Next, we propagate these 12-month surface and volume atlases to each month *τ*_0_ via parallel transport of intra-subject deformation fields estimated using affine and ANTs registration. Warped intensity images are weighted-averaged to obtain the T1w and T2w atlases.

### Reporting summary

Further information on research design is available in the Nature Portfolio Reporting Summary linked to this article.

## Online content

Any methods, additional references, Nature Portfolio reporting summaries, source data, extended data, supplementary information, acknowledgements, peer review information; details of author contributions and competing interests; and statements of data and code availability are available at 10.1038/s41592-022-01703-z.

## Supplementary information

Supplementary Information Supplementary Figs 1–17, Supplementary Tables 1–2, Supplementary Notes 1–4, and Supplementary References.

Reporting Summary

Supplementary Data 1 Source data for Supplementary Figure 5.

Supplementary Data 2 Source data for Supplementary Fig. 7.

Supplementary Data 3 Source data for Supplementary Fig. 8.

Supplementary Data 4 Source data for Supplementary Fig. 10.

Supplementary Data 5 Source data for supplementary Fig. 12.

Supplementary Data 6 Source data for supplementary Fig. 13.

Supplementary Data 7 Source data for supplementary Fig. 17.

## Data Availability

The infant brain atlases are available at Zenodo (10.5281/zenodo.7044932) under the Creative Commons Attribution Non Commercial Share Alike 4.0 International license. The MRI data used in this work can be obtained from the National Institute of Mental Health Data Archive (https://nda.nih.gov/) or by contacting the investigative team^18^. Source data for quantitative results in the Figures 4, 5, and 6, Extended Data Figs. 8 and 10, and Supplementary Figs. 5, 7, 8, 10, 12, 13, and 17 are provided as Excel spreadsheets with this paper.

## Source data

Source Data Fig. 4 Error values, fitted curves, and growth rates.
Source Data Fig. 5 Cortical thickness values of all ROIs across infancy.
Source Data Fig. 6 Cortical T1w/T2w ratio for different cortical lobes/hemispheres.
Source Data Extended Data Fig. 8 Surface area values of all ROIs across infancy.
Source Data Extended Data Fig. 10 Laterality index and statistical tests for cortical thickness.

## Code availability

Software packages used for atlas construction include iBEAT v.2.0, FreeSurfer v.7.2, Spherical Demons v.1.4, FSL v.6.0.5, and RStudio v.1.2.1335. Additional code facilitating atlas construction is available under the MIT license via our project webpage (https://iba.yaplab.io).
