# Supplementary material for: Multifaceted atlases of the human brain in its infancy
Source: Nat Methods. 2022 Dec 30;20(1):55–64. doi: 10.1038/s41592-022-01703-z (PMC9834057; doi:10.1038/s41592-022-01703-z)
Supplement: Supplementary file 1 — Supplementary Figs 1–17, Supplementary Tables 1–2, Supplementary Notes 1–4, and Supplementary References. [file 41592_2022_1703_MOESM1_ESM.pdf]

nature methods

Resource https://doi.org/10.1038/s41592-022-01703-z

Multifaceted atlases of the human brain in its infancy

In the format provided by the authors and unedited

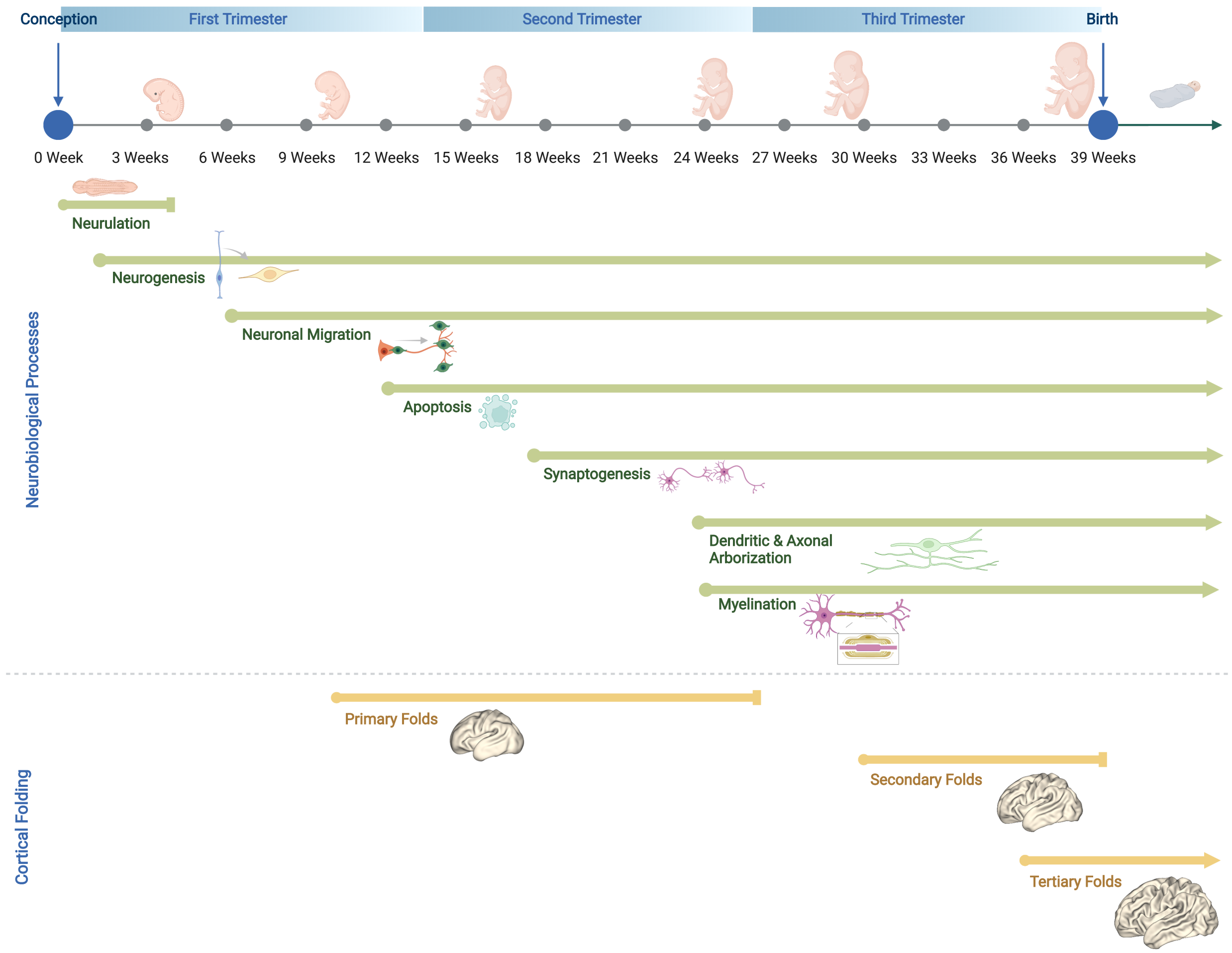

Supplementary Fig. 1 | **Neurodevelopmental timeline.** Schematic illustration of key neurobiological processes[1,2] and cortical folding patterns[3,4] during gestational and perinatal periods.

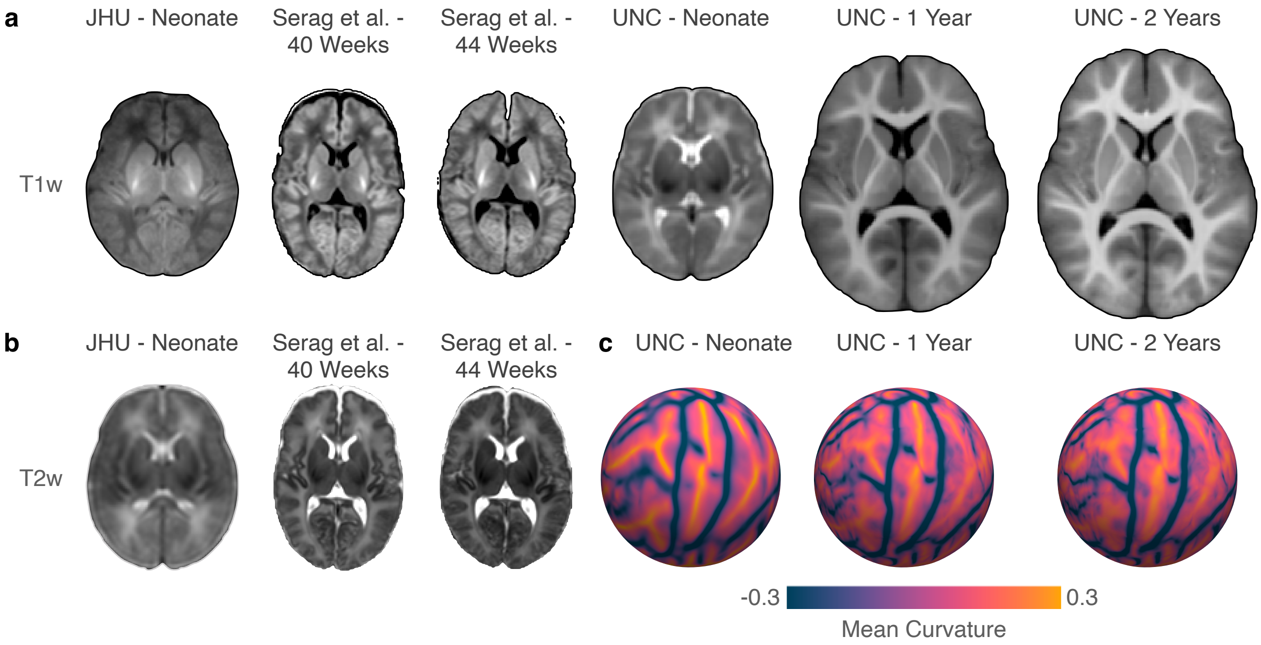

Supplementary Fig. 2 | Existing neonatal and infant atlases. a,**b**, Commonly used T1w and T2w atlases of neonates and infants[5–7]. **c**, Spherical atlases of surface mean curvature of neonates and infants[8].

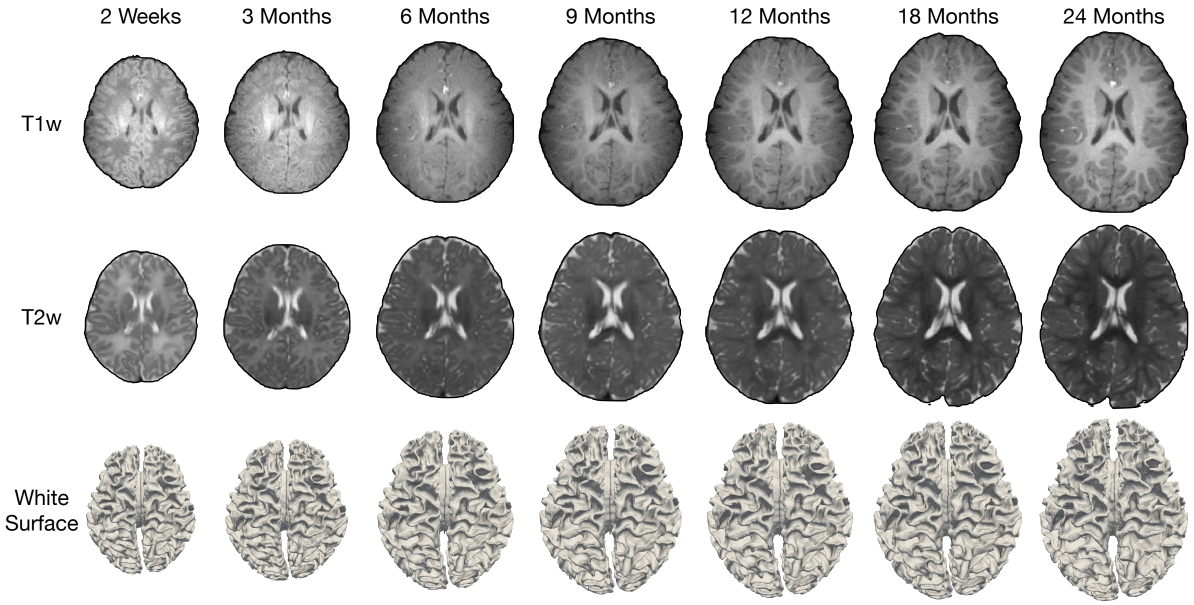

Supplementary Fig. 3 | Longitudinal infant scans. T1w and T2w images and white surfaces of an infant scanned between two weeks and two years of age.

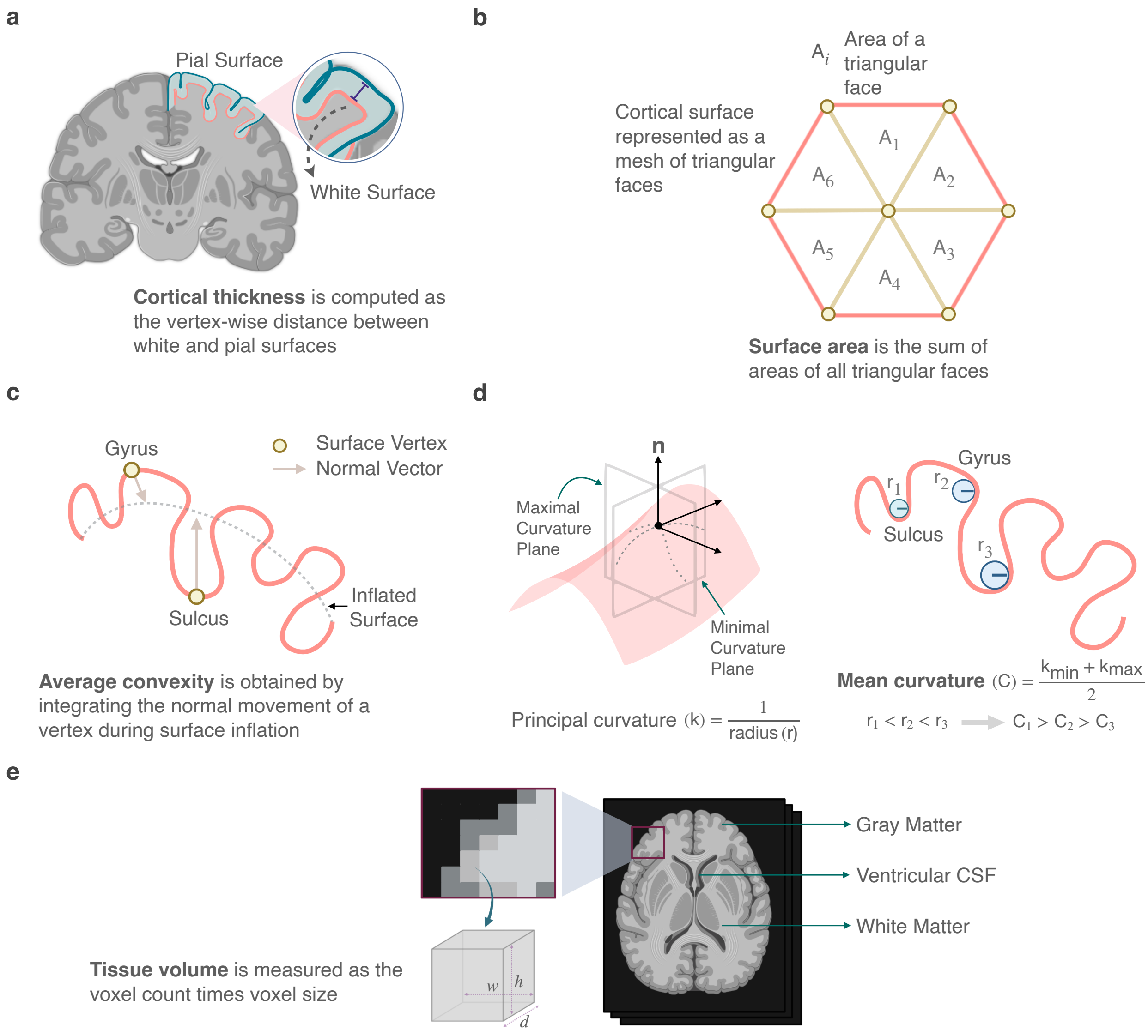

Supplementary Fig. 4 | Surface and volume features. Graphical illustrations of **a**, cortical thickness; **b**, surface area; **c**, average convexity; **d**, mean curvature; and **e**, tissue volume.

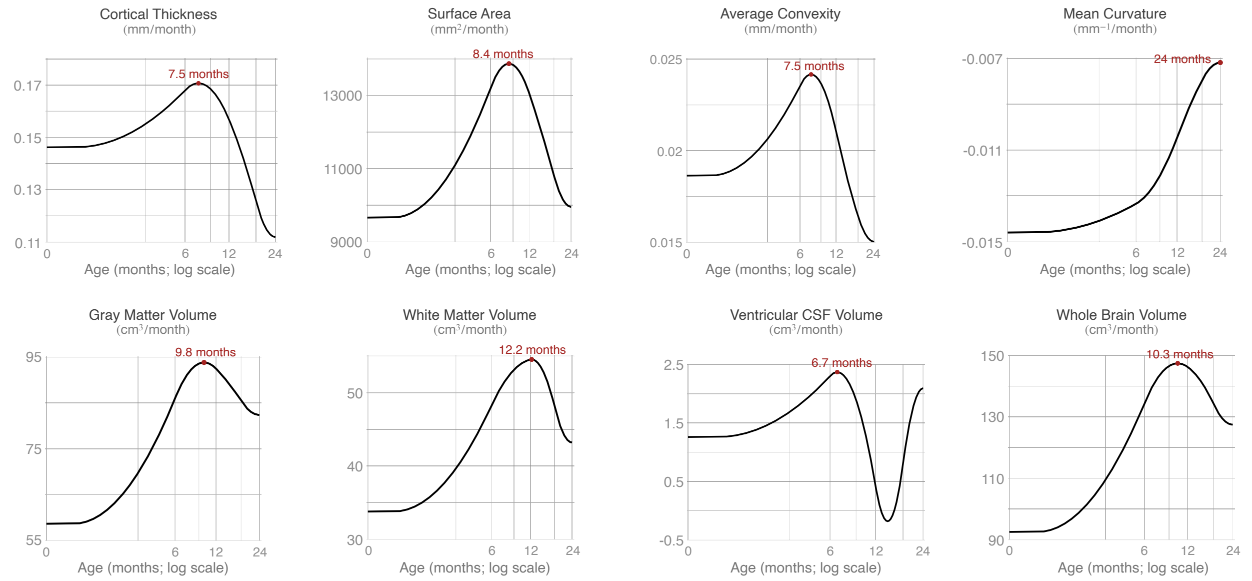

Supplementary Fig. 5 | Cortical and volumetric developmental velocities. IBA velocity curves for surface and volumetric features, estimated via the first derivatives of generalized additive model (GAM)-fitted trajectories. Annotated data points mark the peak growth ages.

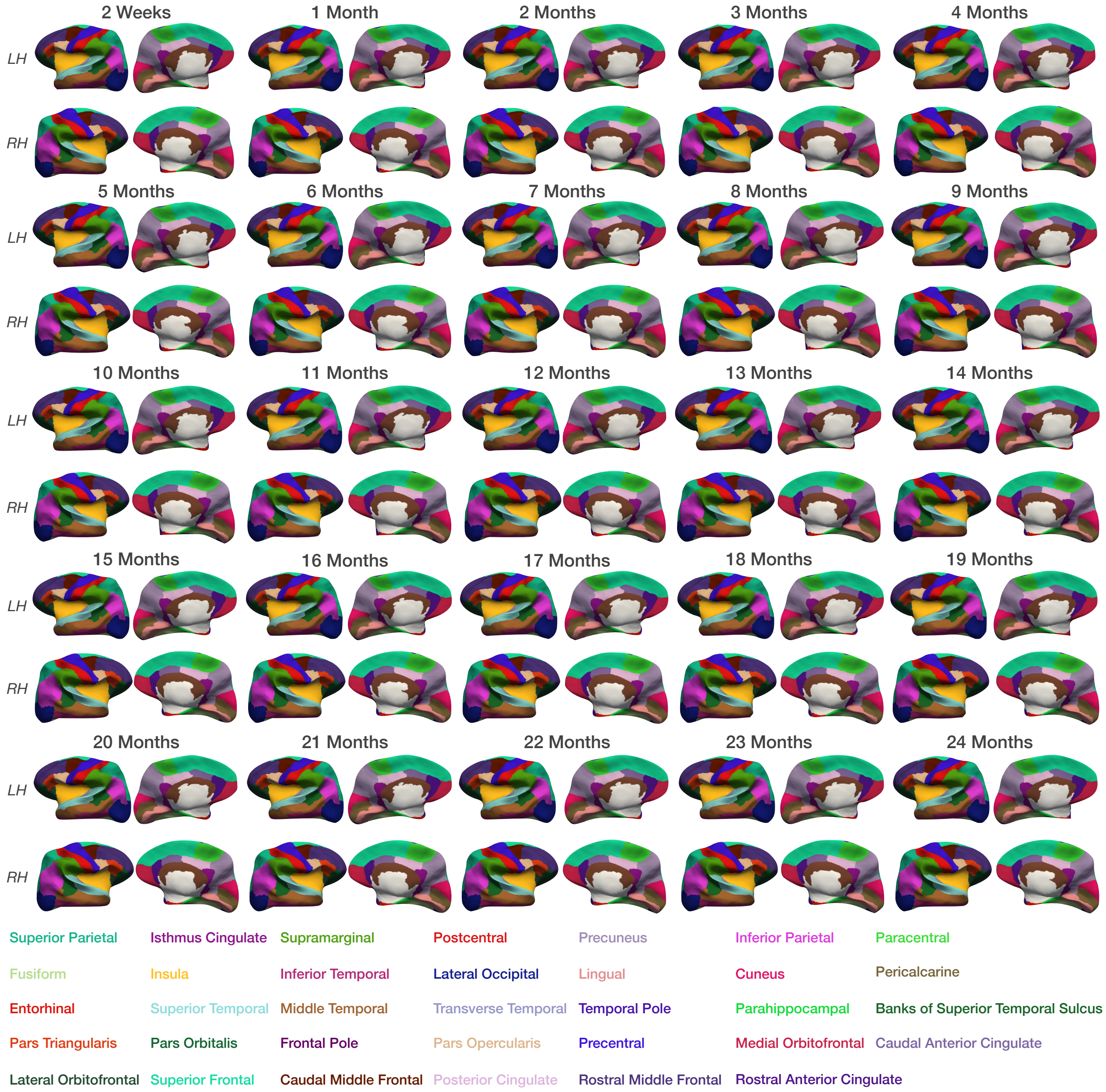

Supplementary Fig. 6 | Cortical ROIs. Desikan-Killiany cortical parcellation mapped onto the inflated white surfaces of the IBA.

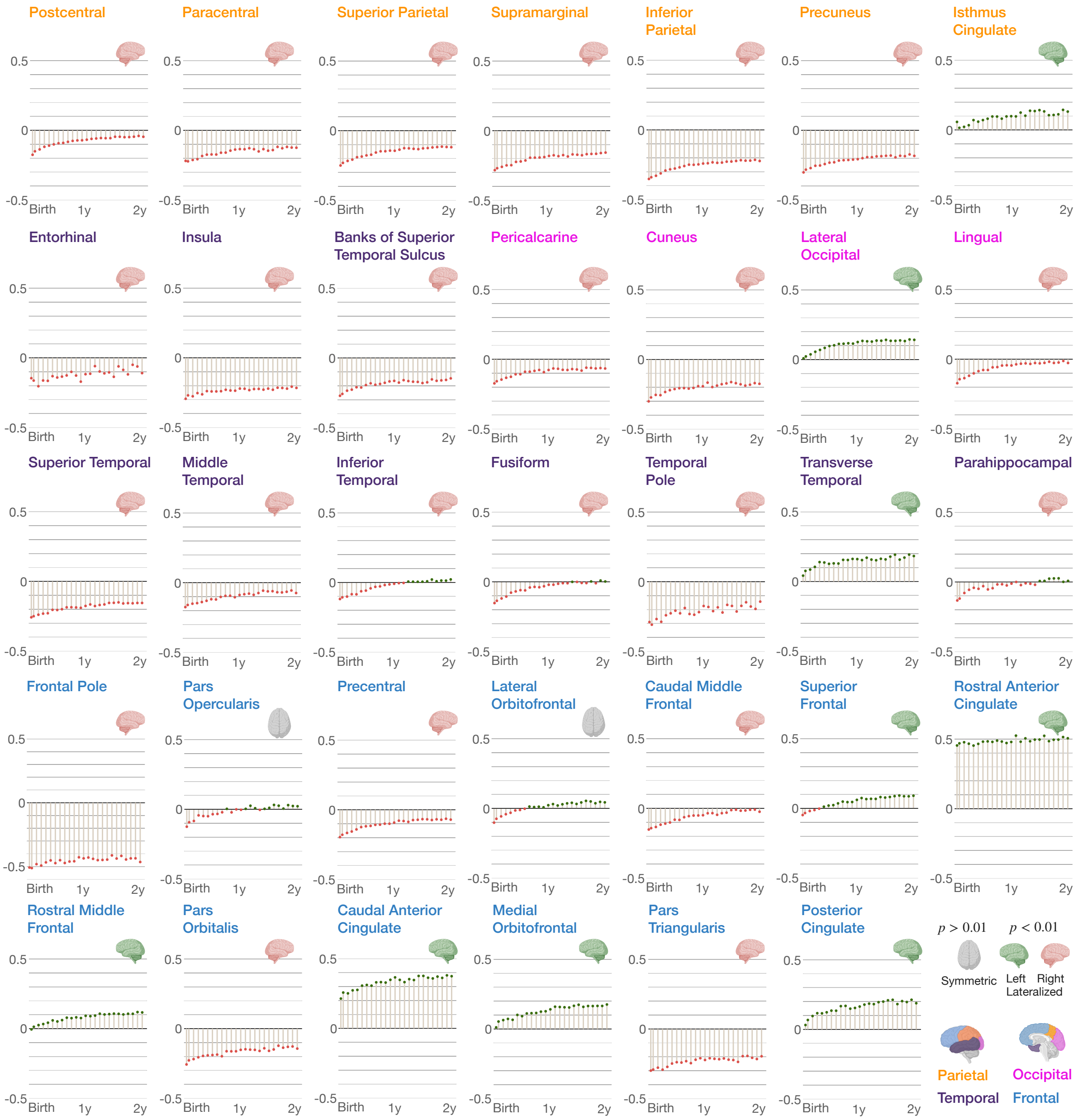

Supplementary Fig. 7 | Hemispheric asymmetry of surface area. Region-specific laterality index for surface area of the IBA. Positive laterality is associated with left lateralization (two-tailed *t*-test: $p < 0.01$) and negative laterality is associated with right lateralization (two-tailed *t*-test: $p < 0.01$).

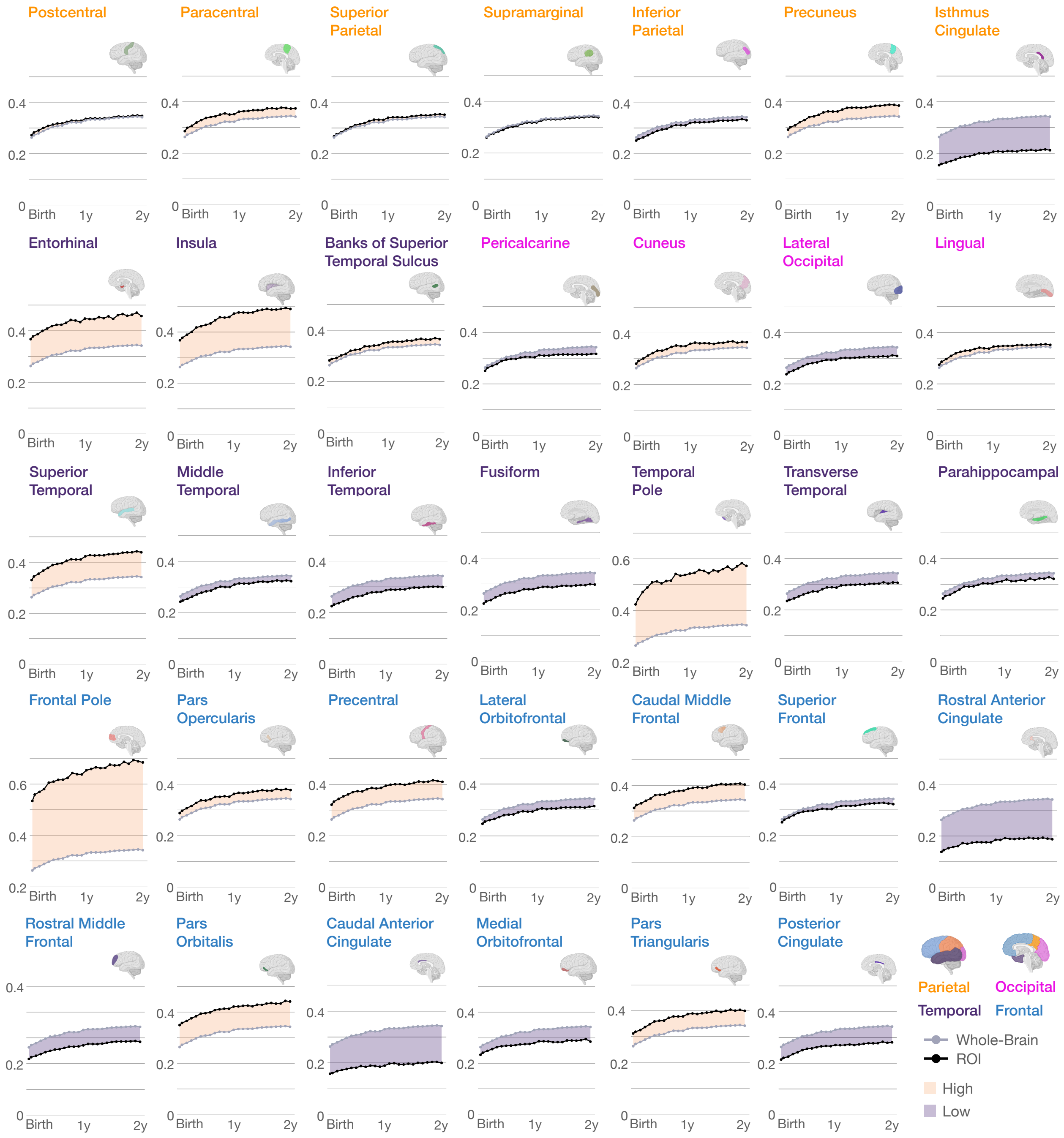

Supplementary Fig. 8 | Regional developmental trajectories of average convexity. Growth curves of average convexity for the IBA cortical regions. Shaded regions indicate whether average convexity is greater or lower than the whole-brain average.

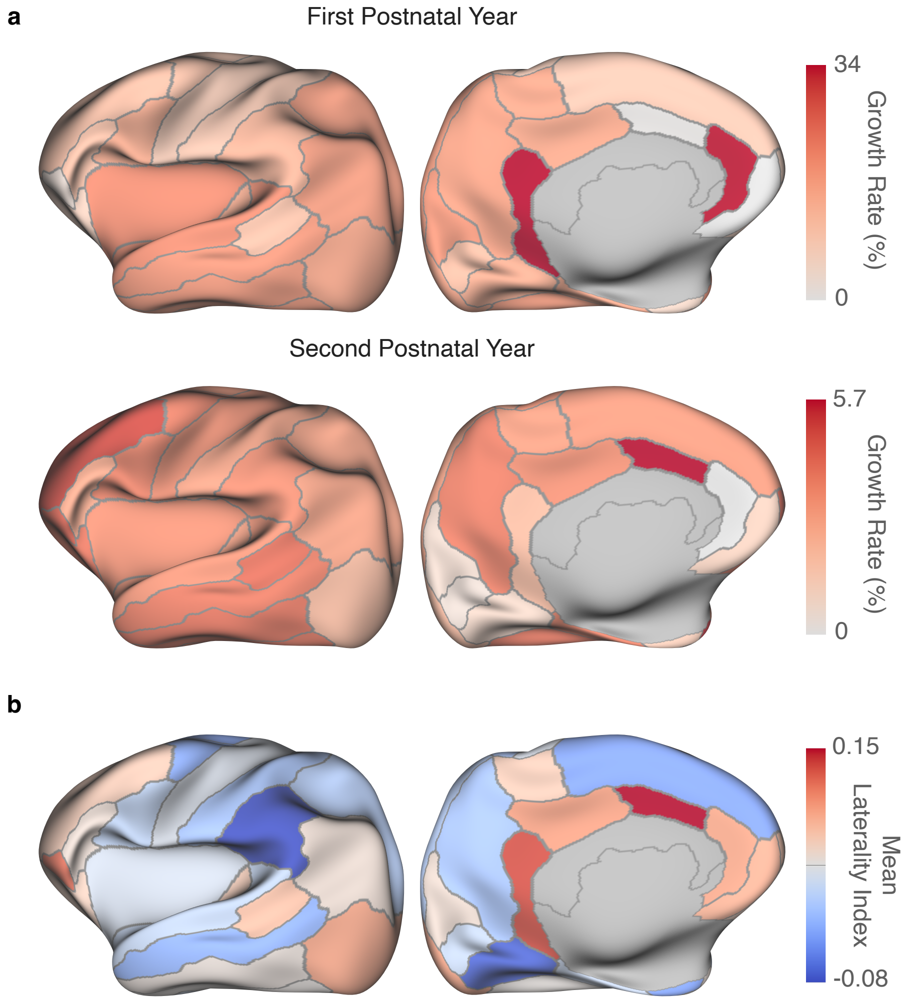

Supplementary Fig. 9 | Analysis of average convexity. **a**, Regional growth rates in terms of average convexity for the first (*top row*) and second (*bottom row*) postnatal years. **b**, ROI-specific mean laterality index for average convexity.

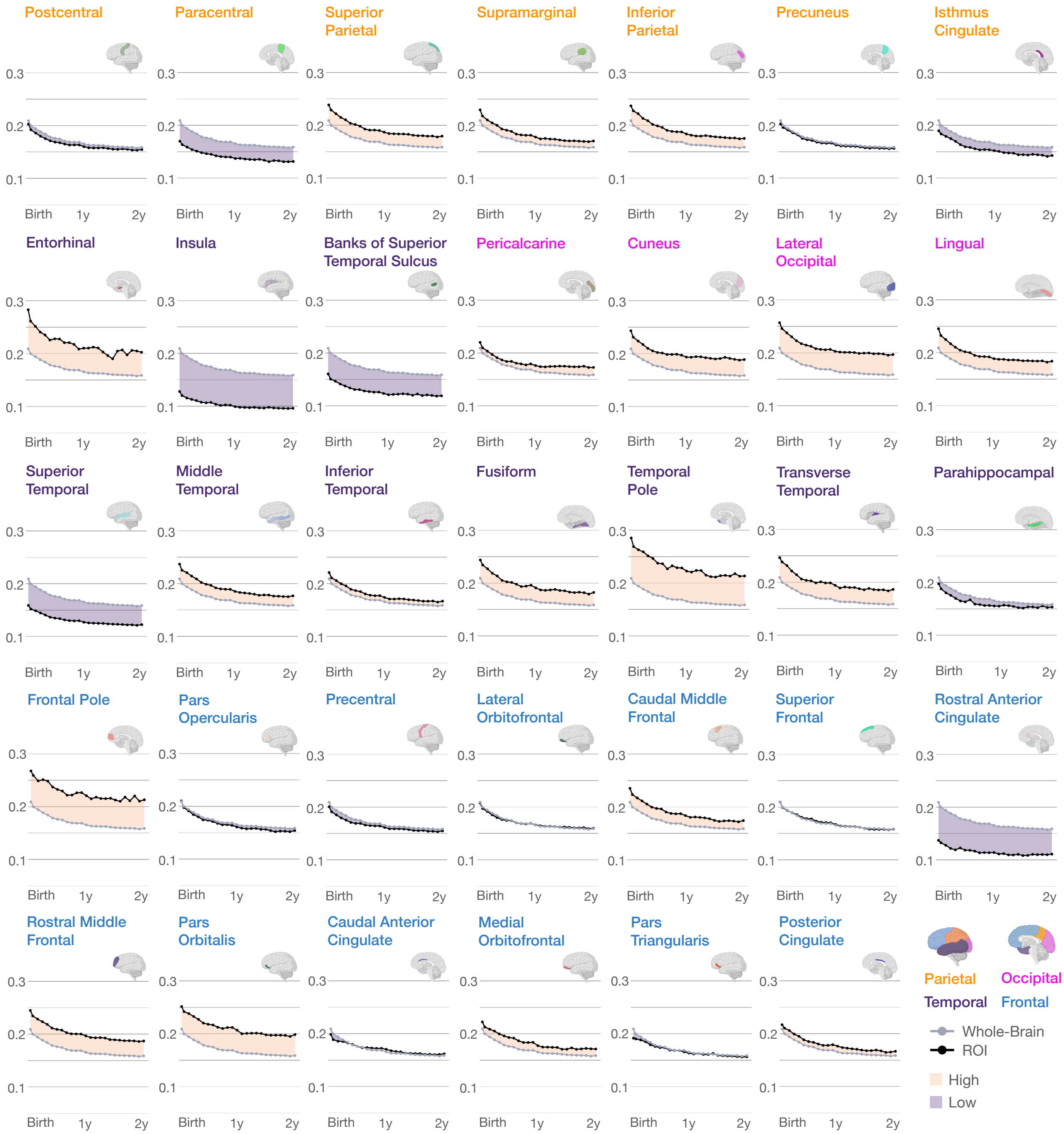

Supplementary Fig. 10 | Regional developmental trajectories of mean curvature. Growth curves of mean curvature for the IBA cortical regions. Shaded regions indicate whether mean curvature is higher or lower than the whole-brain average.

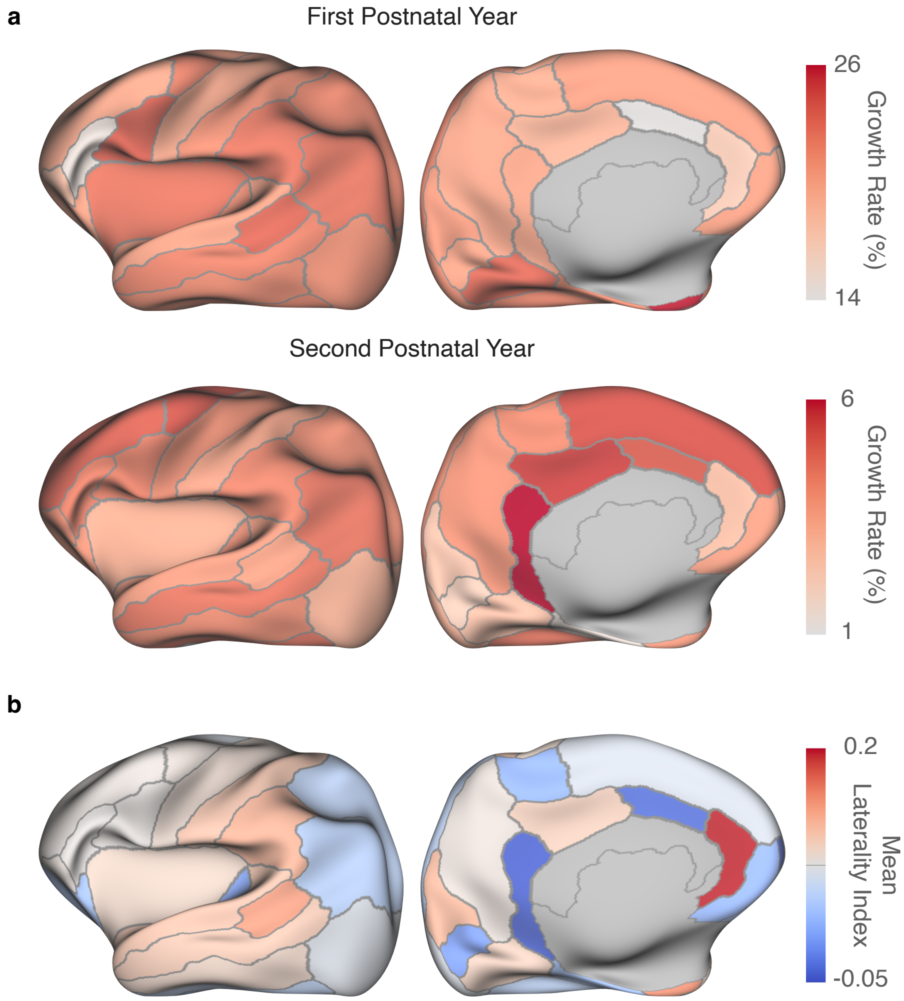

Supplementary Fig. 11 | **Analysis of mean curvature.** **a**, Regional growth rates in terms of mean curvature for the first (*top row*) and second (*bottom row*) postnatal years. **b**, ROI-specific mean laterality index for mean curvature.

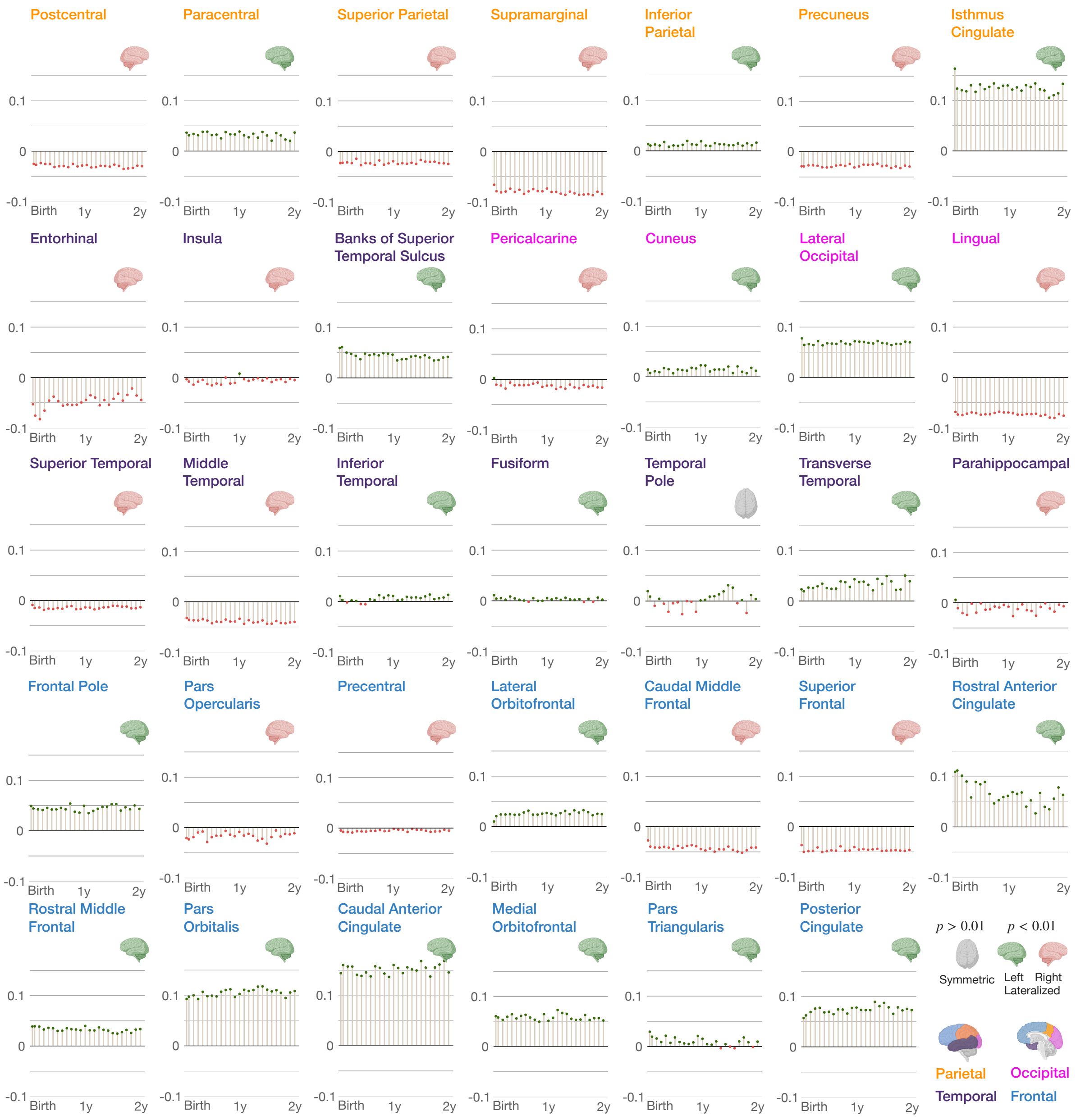

Supplementary Fig. 12 | Hemispheric asymmetry of average convexity. Region-specific laterality index for average convexity of the IBA. Positive laterality is associated with left lateralization (two-tailed t-test: $p < 0.01$) and negative laterality is associated with right lateralization (two-tailed t-test: $p < 0.01$).

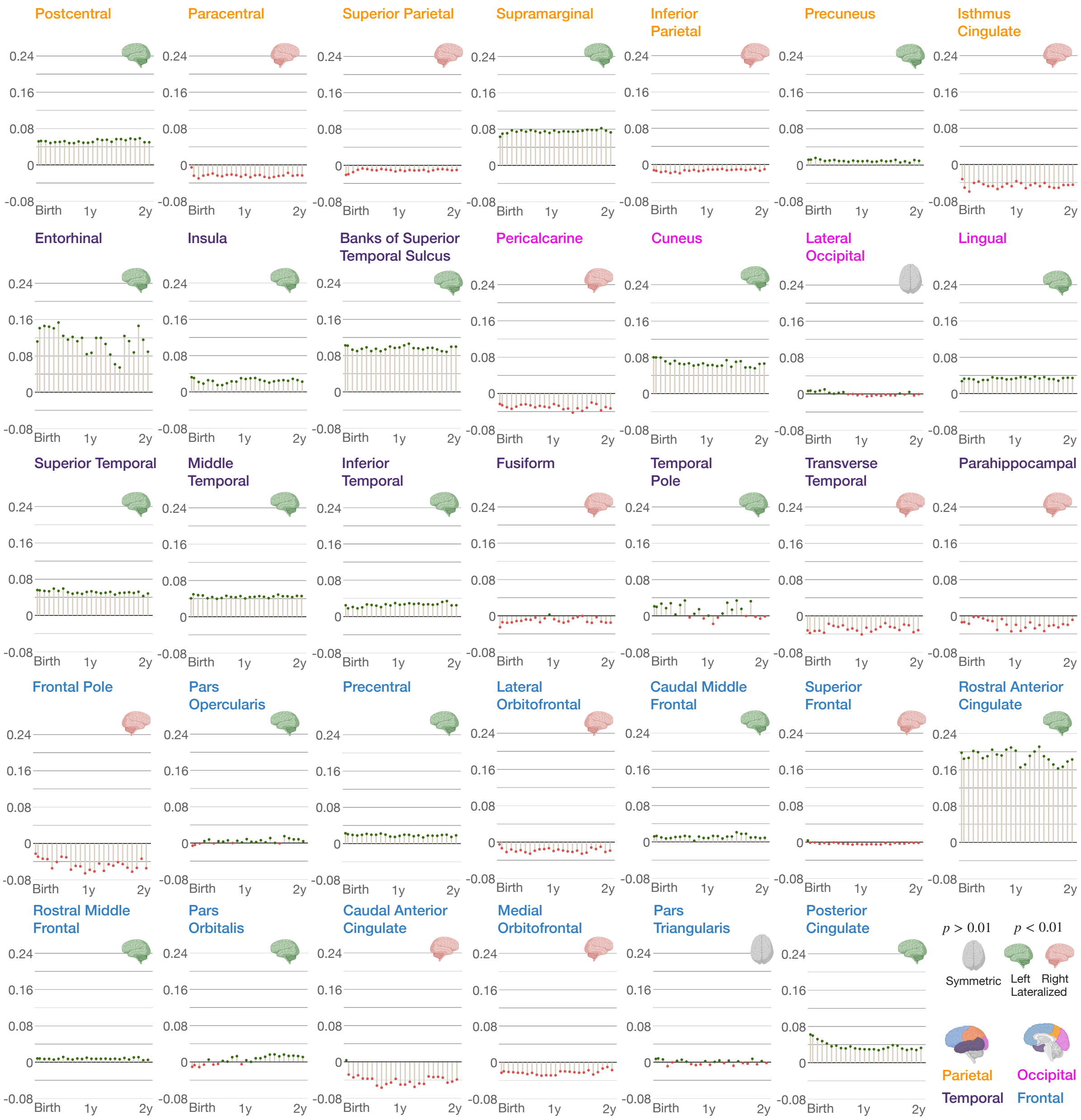

Supplementary Fig. 13 | Hemispheric asymmetry of mean curvature. Region-specific laterality index for mean curvature of the IBA. Positive laterality is associated with left lateralization (two-tailed t-test: $p < 0.01$) and negative laterality is associated with right lateralization (two-tailed t-test: $p < 0.01$).

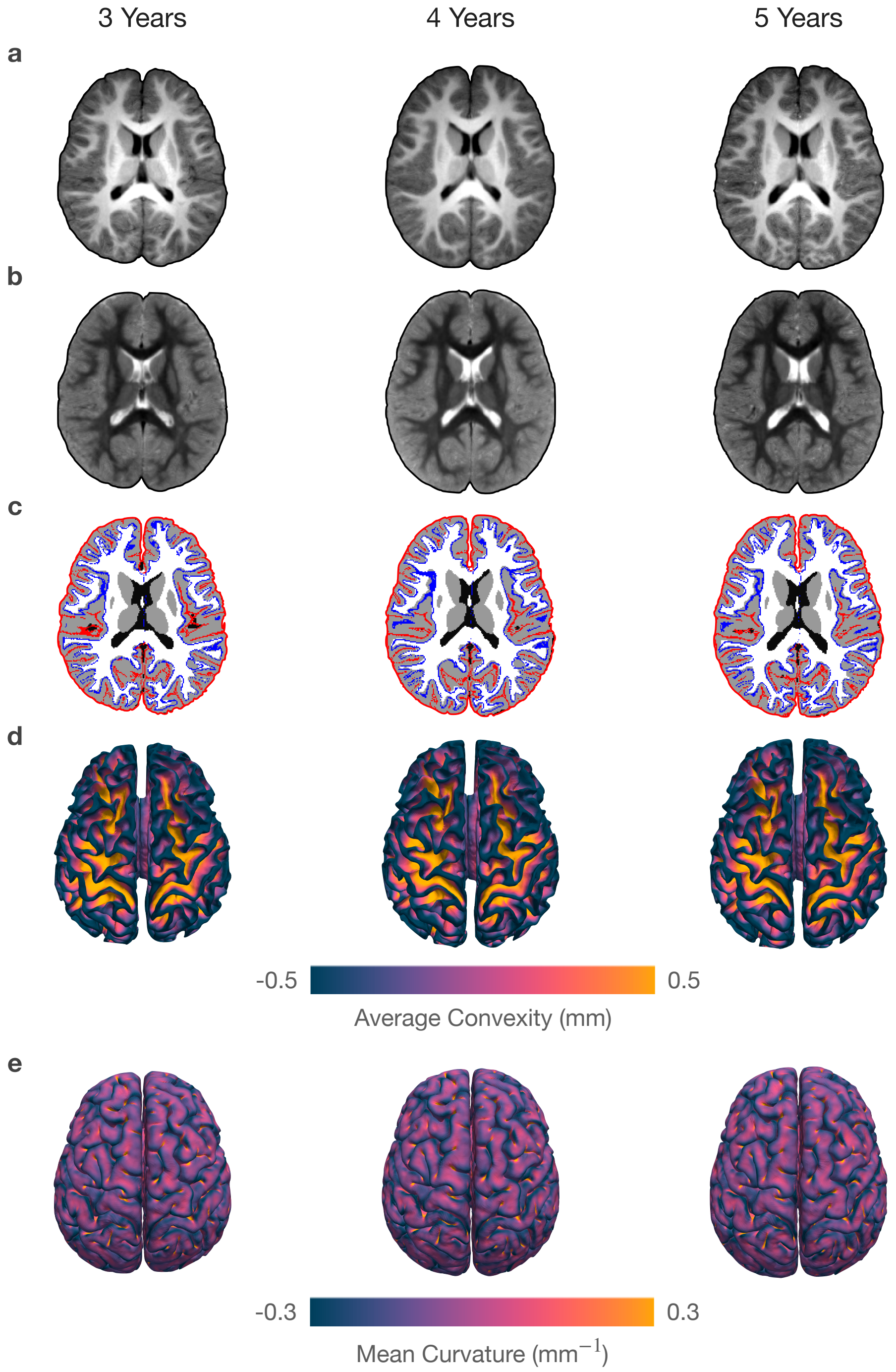

Supplementary Fig. 14 | Preschool atlases. We augment our infant brain atlases with preschool atlases constructed every year between 3 and 5 years of age. These atlases are constructed using MRI data of 49 subjects (recruited as part of the BCP) scanned between 2.5 and 5.5 years of age. We constructed these atlases using our surface-volume atlas construction pipeline. **a**,**b**, Transverse sections of the T1w and T2w atlases. **c**, White (*blue*) and pial (*red*) surface atlases superimposed on the tissue maps. **d**,**e**, Dorsal views of the white and pial surface atlases for both hemispheres, colored by average convexity and mean curvature, respectively.

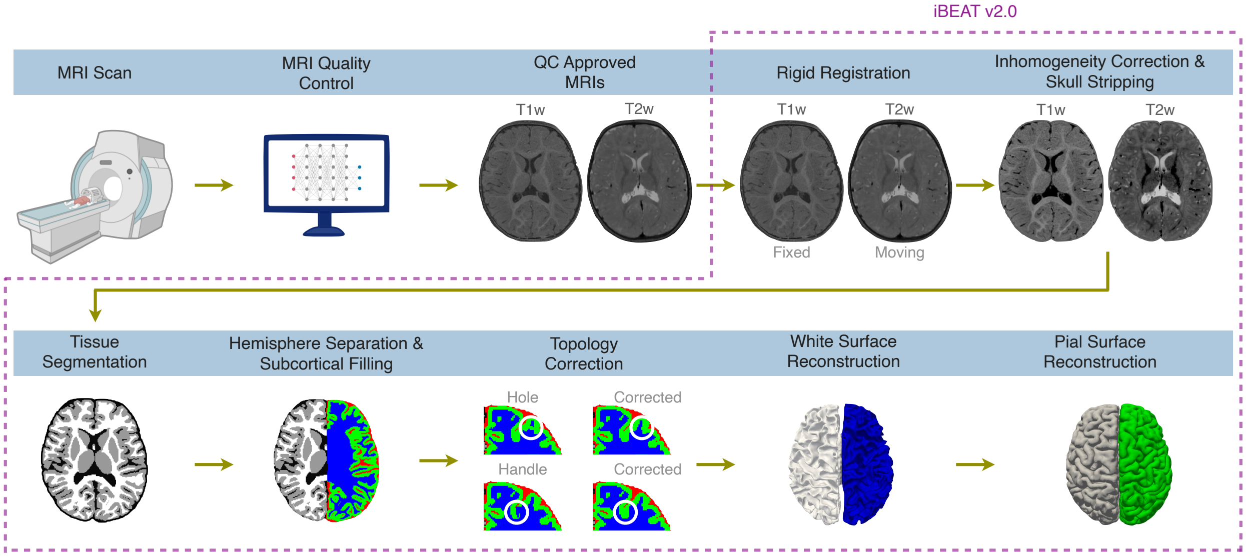

Supplementary Fig. 15 | Infant brain MRI post-acquisition processing. Brain MRI scans quality-checked with our pediatric brain image quality control algorithm[17] are fed into iBEAT v2.0 (`https://ibeat.wildapricot.org`) for infant-centric preprocessing to obtain tissue segmentation maps and white and pial surfaces.

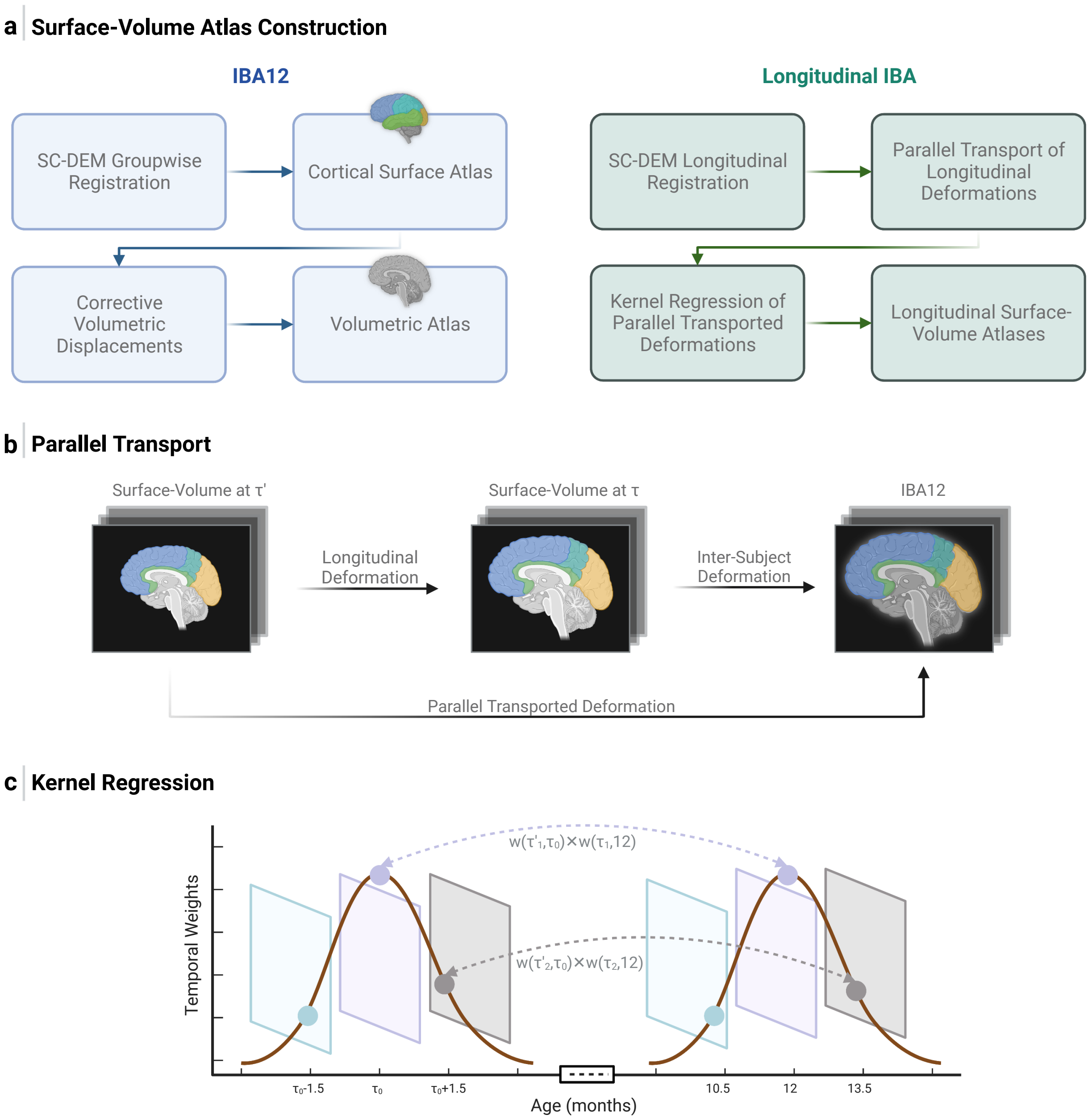

Supplementary Fig. 16 | IBA construction pipeline. **a**, Overview of the steps involved in constructing the surface-volume IBA. **b**, Parallel transport of longitudinal deformations to the IBA12 space. **c**, Weighting of parallel transported deformations.

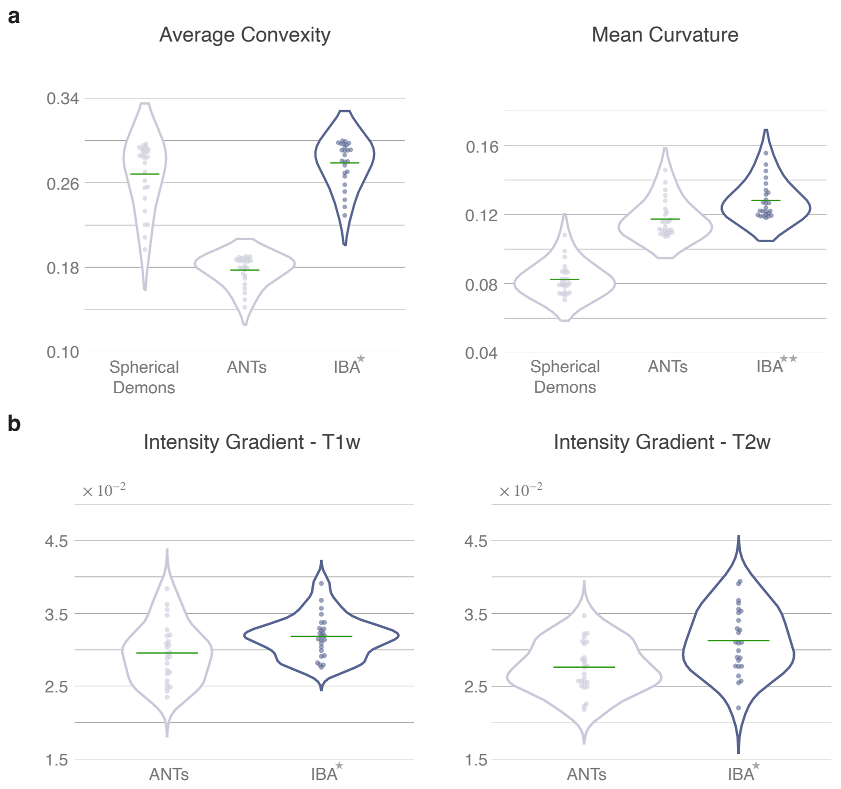

Supplementary Fig. 17 | Quantitative comparison of atlases. **a**, Violin plots for median values of cortical features of the surface atlases. The IBA yields higher absolute average convexity than ANTs (two-tailed paired t-test: $p = 2.9 \times 10^{-29}$; single star) and higher absolute mean curvature than Spherical Demons (two-tailed paired t-test: $p = 1.1 \times 10^{-17}$; double star) and ANTs (two-tailed paired t-test: $p = 1.3 \times 10^{-34}$; double star). **b**, Violin plots for gradient magnitudes of the T1w and T2w intensity atlases. The IBA yields higher gradient magnitudes than ANTs (two-tailed paired t-test: $p_{\mathrm{T1w}} = 1.0 \times 10^{-9}$, $p_{\mathrm{T2w}} = 1.9 \times 10^{-7}$; star). The green crossbars in the violin plots mark the means.

Supplementary Note 1: Results

Surface atlases across infancy. We assessed the degree of cortical folding of each surface atlas with surface-wide median absolute values of average convexity and mean curvature (Supplementary Fig. 17a). A higher median value signifies greater cortical folding. The average convexity median is comparable for Spherical Demons and IBA because they both capture coarse-scale cortical folds. The mean curvature median for the IBA is the highest, suggesting that the IBA not only captures coarse-scale gyral and sulcal patterns but also retains fine-scale secondary and tertiary cortical folds.

Volumetric atlases across infancy. We assessed the quality of the ANTs intensity atlases and IBA using the gradient magnitude, which quantifies structural sharpness (Supplementary Fig. 17b). The IBA has higher gradient magnitudes than the ANTs atlases and is therefore sharper with more structural details.

Surface and volumetric development. We measured the average convexity of 34 cortical regions-of-interest (ROIs) delineated via FreeSurfer using the Desikan-Killiany atlas[18] (Supplementary Fig. 6). All regions (Supplementary Fig. 8) show increasing trends but at varying rates ranging from 19% to 34% and 1.2% to 5.7% for the first and second postnatal years (Supplementary Fig. 9a), respectively. Average convexity exhibits higher growth rate in association cortices compared with primary cortices. The growth rates (Year 1, Year 2) are (24.2%, 1.5%) for the primary visual cortex, (23.3%, 3.3%) for the primary somatosensory cortex, (22.9%, 3.8%) for the primary motor cortex, (27.0%, 3.6%) for the primary auditory cortex, (27.1%, 4.2%) for the temporal association cortex, (27.1%, 3.6%) for the parietal association cortex, and (23.9%, 3.7%) for the prefrontal association cortex.

In terms of mean curvature, all cortical ROIs exhibit decreasing trends (Supplementary Figs. 10 and 11a) at varying negative growth rates ranging from 14% to 26% and 1% to 6% in the first and second postnatal years, respectively. The negative growth rates (Year 1, Year 2) are (19.9%, 2.0%) for the primary visual cortex, (19.5%, 3.9%) for the primary motor cortex, (21.0%, 3.4%) for the primary somatosensory cortex, (20.6%, 3.7%) for the primary auditory cortex, (21.5%, 3.6%) for the temporal association cortex, (21.2%, 4.1%) for the parietal association cortex, and (18.5%, 4.1%) for the prefrontal association cortex.

We show the hemispheric lateralization of regional cortical features via the laterality index, $\text{LI} = (\text{left} - \text{right})/(\text{left} + \text{right})$, computed for each ROI. There is significant asymmetry (two-tailed t-test; $p < 0.01$) in average convexity (Supplementary Fig. 12) and mean curvature (Supplementary Fig. 13) for majority of the ROIs. The corresponding p-values, t-scores, and degrees of freedom (DoFs) are reported in Supplementary Data Files 5, 6. The ROI-specific mean LI for average convexity and mean curvature are shown in Supplementary Figs. 9b and 11b.

Supplementary Note 2: Discussion

Cortical thickness. The IBA reflects the inter-hemispheric differences in regional cortical thickness: the functionally dominant hemisphere is thinner than the non-dominant counterpart (Ex-

tended Data Fig. 10). For instance, the left pars opercularis is thinner, consistent with the functional dominance of the left hemisphere in language production and phonological processing. In contrast, the right pars triangularis is thinner, although the left hemisphere is functionally dominant. This is because pars triangularis is involved in semantic processing that is still imprecise during the first two postnatal years; therefore, the functionally dominant left hemisphere is thicker. The superior temporal gyrus is involved in auditory processing that is pertinent to language acquisition and is dependent on the left hemisphere. The left superior temporal gyrus in IBA is thinner, in line with the early development of infants' ability to process audio and spoken language during the study period. Visuospatial processing controlled by the supramarginal gyrus, inferior parietal cortex, and precuneus is dominant in the right hemisphere. However, infants do not fully acquire visuospatial processing ability during the first two postnatal years; therefore, these regions in IBA are still thicker in the right hemisphere.

Surface area. The IBA also reflects cortical maturation in terms of cortical surface area. According to the radial unit hypothesis[19], cortical surface area expands during infancy due to the symmetrical proliferation of neural progenitors in the ventricular and subventricular zones[20]. Others speculate that intermediate glial cells, generated in the outer subventricular zone, expand in a fan-like manner, and drive the tangential growth of surface area[21,22]. Prior studies suggest that surface area undergoes a 2- to 4-fold expansion from infancy through adulthood[23]. Postnatal surface area expansion is differential in nature, following regional differences in dendritic length, dendritic spine density, synaptic architecture, and intra-cortical myelination[23–26]. The overexpansion of cortical surface area during early infancy is implicated in the disrupted emergence and refinement of cognitive and behavioral skills, and in developmental, psychiatric, and neurological disorders[21,27,28]. The IBA reveals that cortical surface area increases by 93% during the first two years after birth. Findings from our regional analysis are consistent with existing studies[23,29]: regions with greatest expansion lie in the dorsolateral and medial prefrontal association cortex, lateral temporal, and parietal association cortices, whereas regions with least expansion lie in the occipital cortex, medial temporal cortex, and insula. Areas expanding slowly in the primary and secondary sensory cortices in IBA-S are generally thinner, including the pericalcarine, cuneus, precentral, and postcentral gyri. All regions, except the pars opercularis and lateral orbitofrontal gyrus, in IBA-S exhibit significant lateralization. In line with Remer et al.[29], we found left-lateralization of the medial orbitofrontal gyrus, superior frontal gyrus, and transverse temporal gyrus and right-lateralization of the superior and middle temporal gyri, inferior parietal cortex, precuneus, frontal pole, paracentral gyrus, pericalcarine, pars orbitalis, and pars triangularis.

Average convexity. Several hypotheses have been put forward to explain the mechanics driving gyrification, including axonal tension[30], tangential expansion of the cortical layers relative to sublayers generating stress[31], and biochemical prepatterning of the cortex[32]. Although the sizes and shapes of gyri and sulci vary across individuals, primary gyri and sulci share common orientations and locations[33]. Primary folds are associated with motor and sensory cortices, which mature relatively early even at birth and continue to refine postnatally. The IBA faithfully retains major gyral and sulcal folds.

Average convexity quantifies the degree of folding of primary gyri and sulci. Region-specific growth trajectories of average convexity yielded by the IBA reveal that the temporal

association cortex evolves at the fastest rate, followed by the parietal and frontal cortices. Primary sensorimotor and visual cortices grow at a slower rate. This suggests that the primary cortices are already developed at term birth; therefore, they tend to grow slower compared with association cortices, which are involved in high-order functions and are immature at birth and undergo protracted development[34].

The IBA shows inter-hemispheric differences in regional average convexity. Left-lateralized regions are mainly located in the frontal and temporal cortices, whereas right-lateralized regions are prominent in the parietal cortex. This suggests that anatomical asymmetry may correspond to functional asymmetry. For instance, the triangular and orbital parts of the inferior frontal gyrus are language-related[35,36] and therefore exhibit leftward asymmetry. Similarly, the transverse temporal gyrus is left-lateralized as it is part of the auditory processing network[37]. The frontal pole is responsible for action selection and involves more of the left hemisphere than the right[38]. The lateral occipital cortex and fusiform gyrus, involved in face processing, are left-lateralized[39]. Furthermore, our results confirm that the parietal cortex, comprising supramarginal gyrus, superior parietal cortex, and precuneus, shows right laterality, consistent with the functional dominance of the right hemisphere in visuospatial processing tasks[40]. The postcentral gyrus, which is a part of the somatosensory cortex, and the precentral gyrus, which is involved in voluntary motor movements, show rightward asymmetry[40].

Mean curvature. The human brain buckles and folds at different extents[41]. At a larger scale, it curves into a spheroid split into two hemispheres. At a smaller scale, it carves into primary, secondary, and tertiary gyri and sulci in association with cortical development. The degree of small-scale cortical folding is quantified via mean curvature, which measures the extrinsic curvature of the cortical surface[42]. The IBA shows that the absolute mean curvature decreases substantially during the first year and stabilizes in the second year. The decrease in mean curvature is due to the dynamic cortical folding associated with post-migrational processes characterized by axogenesis, dendritogenesis, synapse initiation, maturation, and pruning of both pyramidal neurons and cortical interneurons[43]. Morphologically, the downward trend in mean curvature reflects the widening of sulci due to the increase in brain size, resulting in sulcal fundi and gyral crowns that curve less sharply. Mean curvature exhibits differential decrease in all cortical ROIs: the entorhinal cortex and temporal pole show fastest decline, whereas the rostral and caudal anterior cingulate cortices show slowest decrease. Asymmetry of mean curvature is significant in all regions, except the pars triangularis and lateral occipital gyrus. In line with Remer et al.[29], the posterior cingulate and rostral anterior cingulate cortices are left-lateralized and the lateral orbitofrontal gyrus is right-lateralized. The pars opercularis exhibits leftward asymmetry, consistent with the functional dominance of the left hemisphere in language.

Cortical myelination. We analyzed cortical myelination through infancy via the T1w/T2w ratio. We observed spatiotemporal changes in myelination of the cerebral cortex throughout the study period, in line with the literature[4]. Highly myelinated regions process information faster than less myelinated regions. The primary visual, motor, and somatosensory cortices are myelinated earlier than the association cortices of the frontal, parietal, and temporal lobes that are involved in higher-order functions, consistent with the functional development of the brain. We also observe a correlation between cortical thickness and myelin content: thicker cortices are lightly myelinated compared with thinner cortices that are heavily myelinated. For instance, the prefrontal

cortex has high cortical thickness and low myelin.

Supplementary Note 3: Applications of Brain Atlases

Human brain atlases are the basis of common coordinate frameworks (CCFs)[44] that enable consistent agglomeration of data from diverse imaging modalities, bridging the study of brain architecture from macroscale (e.g., brain regions and their communication pathways) to microscale (e.g., cyto- and myelo-architectures)[45].

Neuroanatomical features (e.g., regional tissue volumes, cortical thickness, and surface areas) extracted from MRI data of individuals can be mapped onto an atlas for volumetric or surface correspondences, allowing for example features of normal and abnormal subjects to be compared in a common space. Inter-group differences can then be detected with the help of statistical tests. Multimodal regional features extracted with the help of an atlas can also be used to identify atypical brain development by predicting the brain age[46]. The difference between the predicted age and the chronological age reflects premature or delayed development associated with brain disorders. In computational anatomy, neuroimaging data of individuals are mapped to a standard space, typically defined by an atlas, via non-rigid registration methods[47]. The resulting nonlinear transformations can be analyzed via statistical tests to investigate inter-individual shape differences.

Atlases provide a standard anatomical space in which tissues, regions, and structures can be annotated at a voxel or vertex level. Tissue classification information can be propagated from an atlas to individual images for tissue segmentation[48]. Brain regions, which define coherent functional units, can be parcellated in an atlas based on cyto- and myelo-architectures or functional connectivity[49]. The atlas parcellation can be propagated for applications in stereotactic localization and structural/functional connectomics[50]. Dimensionality reduction techniques can be applied to the connectivity profiles determined based on a parcellation map to obtain principal gradients, which encode systematic variations in connectivity[51].

Supplementary Note 4: Atlas Variability

We conducted experiments as described by Yang et al.[52] to investigate the effect of sample size on brain atlas variability. Specifically, we constructed infant brain atlases with $N = \{10, 20, 30\}$ randomly selected subjects. For each N, we performed subject sampling and atlas construction three times ($K = 3$) and quantified atlas variability by calculating surface and volumetric displacements between each pair of brain atlases using three metrics:

1. Mean volumetric displacement, $\bar{D}_{\text{vol}}$, computed as the average of

$$D_{\text{vol}} = \frac{1}{V} \sum_{v=1}^{V} (|d_x^v| + |d_y^v| + |d_z^v|), \tag{1}$$

   over all atlas pairs, where V is the total number of brain voxels and (d_x^v, d_y^v, d_z^v) is the displacement of the v-th voxel of an atlas with respect to its pair.

2. Mean surface displacement, $\bar{D}_{\text{surf}}$, computed as the average of

$$D_{\text{surf}} = \frac{1}{S}\sum_{s=1}^{S}(|t_x^s| + |t_y^s| + |t_z^s|), \tag{2}$$

over all atlas pairs, where S is the total number of surface vertices and (t_x^s, t_y^s, t_z^s) is the displacement of s-th vertex of an atlas with respect to its pair.

3. Mean log-transformed Jacobian determinant, $\bar{D}_{\text{jacobian}}$, computed as the average of

$$D_{\text{jacobian}} = \frac{1}{V}\sum_{v=1}^{V}\log(|\det(J^v)|), \tag{3}$$

over all atlas pairs, where $\det(J^v)$ is the determinant of the Jacobian matrix (J^v) computed from the v-th voxel of the volumetric deformation field of an atlas with respect to its pair.

The results, summarized in Supplementary Table 2, indicate that variability decreases with sample size and reaches a subvoxel level at $N = 30$. This confirms that 37 subjects are sufficient for atlas construction.

Supplementary Table 1. Existing neonatal and infant brain atlases.

Study	Type	Components	Age Range	No. of Time Points
Kuklisova *et al.*[9] (2011)	Volumetric	T2w<br>Tissue map	29 - 44 weeks GA	6
Serag *et al.*[6] (2012)	Volumetric	T1w & T2w	27 - 44 weeks PMA	9
Shi *et al.*[7] (2011)	Volumetric	T1w & T2w<br>Tissue map	0 - 2 years	3
Sanchez *et al.*[10] (2012)	Volumetric	T1w & T2w	2 weeks - 4 years	13
Zhang *et al.*[11] (2016)	Volumetric	T1w & T2w	1 - 12 months	5
Oishi *et al.*[5] (2011)	Volumetric	T1w & T2w<br>DTI	2 days	1
Kazemi *et al.*[12] (2007)	Volumetric	T1w	39 - 42 weeks GA	1
Shi *et al.*[13] (2010)	Volumetric	T2w<br>Tissue map	0.6 - 2 months	1
Hashioka *et al.*[14] (2011)	Volumetric	T2w	1 week	1
Bozek *et al.*[4] (2018)	Surface	Fiducial cortical surfaces<br>Cortical features maps	36 - 44 weeks PMA	9
Hill *et al.*[15] (2010)	Surface	Spherical cortical surfaces<br>Fiducial cortical surfaces<br>Sulcal depth map	$>$ 36 weeks GA	1
Wu *et al.*[8] (2019)	Surface	Spherical cortical surfaces<br>Cortical features maps<br>Cortical parcellation	1 - 72 months	11
Li *et al.*[16] (2015)	Surface	Spherical cortical surfaces<br>Cortical features maps	1 - 24 months	7

GA – gestational age PMA – postmenstrual age DTI – diffusion tensor imaging

Supplementary Table 2. The effects of sample size (N) on atlas variability quantified via mean volumetric displacement ($\bar{D}_{\text{vol}}$), mean surface displacement ($\bar{D}_{\text{surf}}$), and mean log-transformed Jacobian determinant ($\bar{D}_{\text{jacobian}}$).

N	$\bar{D}_{\text{vol}}$ (mm)	$\bar{D}_{\text{surf}}$ (mm)	$\bar{D}_{\text{jacobian}}$
10	0.6	1.5	0.06
20	0.4	0.8	0.04
30	0.09	0.4	0.01

Supplementary References

1. Stiles, J. & Jernigan, T. L. The basics of brain development. *Neuropsychology Review* **20**, 327 – 348 (2010). URL `https://doi.org/10.1007/s11065-010-9148-4`.

2. Graham, A. M., Marr, M., Buss, C., Sullivan, E. L. & Fair, D. A. Understanding vulnerability and adaptation in early brain development using network neuroscience. *Trends in Neurosciences* **44**, 276 – 288 (2021). URL `https://doi.org/10.1016/j.tins.2021.01.008`.

3. Chi, J. G., Dooling, E. C. & Gilles, F. H. Gyral development of the human brain. *Annals of Neurology* **1**, 86 – 93 (1977). URL `https://doi.org/10.1002/ana.410010109`.

4. Bozek, J. *et al.* Construction of a neonatal cortical surface atlas using multimodal surface matching in the developing human connectome project. *NeuroImage* **179**, 11 – 29 (2018). URL `https://doi.org/10.1016/j.neuroimage.2018.06.018`.

5. Oishi, K. *et al.* Multi-contrast human neonatal brain atlas: Application to normal neonate development analysis. *NeuroImage* **56**, 8 – 20 (2011). URL `http://www.sciencedirect.com/science/article/pii/S1053811911000875`.

6. Serag, A. *et al.* Construction of a consistent high-definition spatio-temporal atlas of the developing brain using adaptive kernel regression. *NeuroImage* **59**, 2255 – 2265 (2012). URL `https://doi.org/10.1016/j.neuroimage.2011.09.062`.

7. Shi, F. *et al.* Infant brain atlases from neonates to 1- and 2-year-olds. *PLOS ONE* **6**, 1 – 11 (2011). URL `https://doi.org/10.1371/journal.pone.0018746`.

8. Wu, Z. *et al.* Construction of 4D infant cortical surface atlases with sharp folding patterns via spherical patch-based group-wise sparse representation. *Human Brain Mapping* **40**, 3860 – 3880 (2019). URL `https://onlinelibrary.wiley.com/doi/abs/10.1002/hbm.24636`.

9. Kuklisova-Murgasova, M. *et al.* A dynamic 4D probabilistic atlas of the developing brain. *NeuroImage* **54**, 2750 – 2763 (2011). URL `https://doi.org/10.1016/j.neuroimage.2010.10.019`.

10. Sanchez, C. E., Richards, J. E. & Almli, C. R. Neurodevelopmental MRI brain templates for children from 2 weeks to 4 years of age. *Developmental Psychobiology* **54**, 77 – 91 (2012). URL `https://doi.org/10.1523/10.1002/dev.20579`.

11. Zhang, Y. *et al.* Consistent spatial-temporal longitudinal atlas construction for developing infant brains. *IEEE Transactions on Medical Imaging* **35**, 2568 – 2577 (2016). URL `https://doi.org/10.1109/TMI.2016.2587628`.

12. Kazemi, K., Moghaddam, H. A., Grebe, R., Gondry-Jouet, C. & Wallois, F. A neonatal atlas template for spatial normalization of whole-brain magnetic resonance images of newborns: Preliminary results. *NeuroImage* **37**, 463 – 473 (2007). URL `http://www.sciencedirect.com/science/article/pii/S1053811907004284`.

13. Shi, F. *et al.* Construction of multi-region-multi-reference atlases for neonatal brain MRI segmentation. *NeuroImage* **51**, 684 – 693 (2010). URL https://www.sciencedirect.com/science/article/pii/S105381191000193X.

14. Hashioka, A. *et al.* A neonatal brain MR image template of 1 week newborn. *International Journal of Computer Assisted Radiology and Surgery* **7**, 273 – 280 (2011). URL https://doi.org/10.1007/s11548-011-0646-5.

15. Hill, J. *et al.* A surface-based analysis of hemispheric asymmetries and folding of cerebral cortex in term-born human infants. *Journal of Neuroscience* **30**, 2268 – 2276 (2010). URL https://doi.org/10.1523/JNEUROSCI.4682-09.2010.

16. Li, G. *et al.* Construction of 4D high-definition cortical surface atlases of infants: Methods and applications. *Medical Image Analysis* **25**, 22 – 36 (2015). URL https://doi.org/10.1016/j.media.2015.04.005.

17. Liu, S., Thung, K.-H., Lin, W., Yap, P.-T. & Shen, D. Real-time quality assessment of pediatric MRI via semi-supervised deep nonlocal residual neural networks. *IEEE Transactions on Image Processing* **29**, 7697 – 7706 (2020). URL https://doi.org/10.1109/TIP.2020.2992079.

18. Fischl, B. Freesurfer. *NeuroImage* **62**, 774 – 781 (2012). URL https://doi.org/10.1016/j.neuroimage.2012.01.021.

19. Rakic, P. Radial versus tangential migration of neuronal clones in the developing cerebral cortex. *Proceedings of the National Academy of Sciences* **92**, 11323 – 11327 (1995). URL https://www.pnas.org/content/92/25/11323.

20. Im, K. & Grant, P. E. Sulcal pits and patterns in developing human brains. *NeuroImage* **185**, 881 – 890 (2019). URL https://www.sciencedirect.com/science/article/pii/S1053811918302672.

21. Piven, J., Elison, J. T. & Zylka, M. J. Toward a conceptual framework for early brain and behavior development in autism. *Molecular Psychiatry* **22**, 1385 – 1394 (2017). URL https://doi.org/10.1038/mp.2017.131.

22. Reillo, I., de Juan Romero, C., García-Cabezas, M. A. & Borrell, V. A role for intermediate radial glia in the tangential expansion of the mammalian cerebral cortex. *Cerebral Cortex* **21**, 1674 – 1694 (2010). URL https://doi.org/10.1093/cercor/bhq238.

23. Hill, J. *et al.* Similar patterns of cortical expansion during human development and evolution. *Proceedings of the National Academy of Sciences* **107**, 13135 – 13140 (2010). URL https://www.pnas.org/content/107/29/13135.

24. Cafiero, R., Brauer, J., Anwander, A. & Friederici, A. D. The concurrence of cortical surface area expansion and white matter myelination in human brain development. *Cerebral Cortex* **29**, 827 – 837 (2018). URL https://doi.org/10.1093/cercor/bhy277.

25. Travis, K., Ford, K. & Jacobs, B. Regional dendritic variation in neonatal human cortex: a quantitative Golgi study. *Developmental Neuroscience* **27**, 277 – 287 (2005). URL https://doi.org/10.1159/000086707.

26. Huttenlocher, P. R. & Dabholkar, A. S. Regional differences in synaptogenesis in human cerebral cortex. *Journal of Comparative Neurology* **387**, 167 – 178 (1997). URL https://onlinelibrary.wiley.com/doi/abs/10.1002/.

27. Rapoport, J. L., Giedd, J. N. & Gogtay, N. Neurodevelopmental model of schizophrenia: update 2012. *Molecular Psychiatry* **17**, 1228 – 1238 (2012). URL https://doi.org/10.1038/mp.2012.23.

28. Rimol, L. M. *et al.* Cortical volume, surface area, and thickness in schizophrenia and bipolar disorder. *Biological Psychiatry* **71**, 552 – 560 (2012). URL https://www.sciencedirect.com/science/article/pii/S0006322311011991. Translational Neuroscience Insights into Neuroplasticity Deficits in Schizophrenia.

29. Remer, J. *et al.* Quantifying cortical development in typically developing toddlers and young children, 1-6 years of age. *NeuroImage* **153**, 246 – 261 (2017). URL https://www.sciencedirect.com/science/article/pii/S1053811917302999.

30. Essen, D. C. V. A tension-based theory of morphogenesis and compact wiring in the central nervous system. *Nature* **385**, 313 – 318 (1997). URL https://doi.org/10.1038/385313a0.

31. Ronan, L. *et al.* Differential tangential expansion as a mechanism for cortical gyrification. *Cerebral Cortex* **24**, 2219 – 2228 (2013). URL https://doi.org/10.1093/cercor/bht082.

32. Welker, W. *Why does cerebral cortex fissure and fold?*, 3 – 136 (Springer US, Boston, MA, 1990). URL https://doi.org/10.1007/978-1-4615-3824-0_1.

33. Dubois, J. *et al.* Primary cortical folding in the human newborn: an early marker of later functional development. *Brain* **131**, 2028 – 2041 (2008). URL https://doi.org/10.1093/brain/awn137.

34. Casey, B., Tottenham, N., Liston, C. & Durston, S. Imaging the developing brain: what have we learned about cognitive development? *Trends in Cognitive Sciences* **9**, 104 – 110 (2005). URL https://www.sciencedirect.com/science/article/pii/S1364661305000306. Special issue: Developmental cognitive neuroscience.

35. Kong, X.-Z. *et al.* Mapping cortical brain asymmetry in 17,141 healthy individuals worldwide via the ENIGMA consortium. *Proceedings of the National Academy of Sciences* **115**, E5154 – E5163 (2018). URL https://www.pnas.org/content/115/22/E5154.

36. Bishop, D. V. M. Cerebral asymmetry and language development: Cause, correlate, or consequence? *Science* **340**, 1230531 (2013). URL https://www.science.org/doi/abs/10.1126/science.1230531.

37. Devlin, J. T. *et al.* Functional asymmetry for auditory processing in human primary auditory cortex. *Journal of Neuroscience* **23**, 11516 – 11522 (2003). URL https://www.jneurosci.org/content/23/37/11516.

38. Goel, V., Shuren, J., Sheesley, L. & Grafman, J. Asymmetrical involvement of frontal lobes in social reasoning. *Brain* **127**, 783 – 790 (2004). URL https://doi.org/10.1093/brain/awh086.

39. Meng, M., Cherian, T., Singal, G. & Sinha, P. Lateralization of face processing in the human brain. *Proceedings of the Royal Society B: Biological Sciences* **279**, 2052 – 2061 (2012). URL https://royalsocietypublishing.org/doi/abs/10.1098/rspb.2011.1784.

40. Toga, A. W. & Thompson, P. M. Mapping brain asymmetry. *Nature Reviews Neuroscience* **4**, 37 – 48 (2003). URL https://doi.org/10.1038/nrn1009.

41. Pienaar, R., Fischl, B., Caviness, V., Makris, N. & Grant, P. E. A methodology for analyzing curvature in the developing brain from preterm to adult. *International Journal of Imaging Systems and Technology* **18**, 42 – 68 (2008). URL https://onlinelibrary.wiley.com/doi/abs/10.1002/ima.20138.

42. Deppe, M. *et al.* Increased cortical curvature reflects white matter atrophy in individual patients with early multiple sclerosis. *NeuroImage: Clinical* **6**, 475 – 487 (2014). URL https://www.sciencedirect.com/science/article/pii/S2213158214000308.

43. Severino, M. *et al.* Definitions and classification of malformations of cortical development: practical guidelines. *Brain* **143**, 2874 – 2894 (2020). URL https://doi.org/10.1093/brain/awaa174.

44. Rood, J. E. *et al.* Toward a common coordinate framework for the human body. *Cell* **179**, 1455 – 1467 (2019). URL https://doi.org/10.1016/j.cell.2019.11.019.

45. van den Heuvel, M. P. & Yeo, B. T. A spotlight on bridging microscale and macroscale human brain architecture. *Neuron* **93**, 1248 – 1251 (2017). URL https://doi.org/10.1016/j.neuron.2017.02.048.

46. Wang, Q. *et al.* Predicting brain age during typical and atypical development based on structural and functional neuroimaging. *Human Brain Mapping* **42**, 5943 – 5955 (2021). URL https://doi.org/10.1002/hbm.25660.

47. Joshi, S., Davis, B., Jomier, M. & Gerig, G. Unbiased diffeomorphic atlas construction for computational anatomy. *NeuroImage* **23**, S151 – S160 (2004). URL https://doi.org/10.1016/j.neuroimage.2004.07.068. Mathematics in Brain Imaging.

48. Aljabar, P., Heckemann, R., Hammers, A., Hajnal, J. & Rueckert, D. Multi-atlas based segmentation of brain images: Atlas selection and its effect on accuracy. *NeuroImage* **46**, 726 – 738 (2009). URL https://doi.org/10.1016/j.neuroimage.2009.02.018.

49. Evans, A. C., Janke, A. L., Collins, D. L. & Baillet, S. Brain templates and atlases. *NeuroImage* **62**, 911 – 922 (2012). URL https://doi.org/10.1016/j.neuroimage.2012.01.024.

50. Rohlfing, T., Zahr, N. M., Sullivan, E. V. & Pfefferbaum, A. The SRI24 multichannel atlas of normal adult human brain structure. *Human Brain Mapping* **31**, 798 – 819 (2010).

51. Seidlitz, J. *et al.* Morphometric similarity networks detect microscale cortical organization and predict inter-individual cognitive variation. *Neuron* **97**, 231 – 247.e7 (2018). URL https://doi.org/10.1016/j.neuron.2017.11.039.

52. Yang, G. *et al.* Sample sizes and population differences in brain template construction. *NeuroImage* **206**, 116318 (2020). URL https://doi.org/10.1016/j.neuroimage.2019.116318.
