# Supplementary material for: Multifaceted atlases of the human brain in its infancy
Source: Nat Methods. 2022 Dec 30;20(1):55–64. doi: 10.1038/s41592-022-01703-z (PMC9834057; doi:10.1038/s41592-022-01703-z)
Supplement: Supplementary file 2 — Reporting Summary [file 41592_2022_1703_MOESM2_ESM.pdf]

nature research

Corresponding author(s): Pew-Thian Yap, Ph.D.

Last updated by author(s): Oct 21, 2022

Reporting Summary

Nature Research wishes to improve the reproducibility of the work that we publish. This form provides structure for consistency and transparency in reporting. For further information on Nature Research policies, see our Editorial Policies and the Editorial Policy Checklist.

Statistics

For all statistical analyses, confirm that the following items are present in the figure legend, table legend, main text, or Methods section.

n/a	Confirmed	
☐	☒	The exact sample size (n) for each experimental group/condition, given as a discrete number and unit of measurement
☐	☒	A statement on whether measurements were taken from distinct samples or whether the same sample was measured repeatedly
☐	☒	The statistical test(s) used AND whether they are one- or two-sided<br>*Only common tests should be described solely by name; describe more complex techniques in the Methods section.*
☒	☐	A description of all covariates tested
☒	☐	A description of any assumptions or corrections, such as tests of normality and adjustment for multiple comparisons
☐	☒	A full description of the statistical parameters including central tendency (e.g. means) or other basic estimates (e.g. regression coefficient) AND variation (e.g. standard deviation) or associated estimates of uncertainty (e.g. confidence intervals)
☐	☒	For null hypothesis testing, the test statistic (e.g. F, t, r) with confidence intervals, effect sizes, degrees of freedom and P value noted<br>*Give P values as exact values whenever suitable.*
☒	☐	For Bayesian analysis, information on the choice of priors and Markov chain Monte Carlo settings
☒	☐	For hierarchical and complex designs, identification of the appropriate level for tests and full reporting of outcomes
☒	☐	Estimates of effect sizes (e.g. Cohen's d, Pearson's r), indicating how they were calculated

Our web collection on statistics for biologists contains articles on many of the points above.

Software and code

Policy information about availability of computer code

Data collection	No software was used
Data analysis	Atlas construction depends on several open-source software packages including: iBEAT v2.0, FreeSurfer v7.2, Spherical Demons v1.4, FSL v6.0.5, and RStudio v1.2.1335. Additional code facilitating atlas construction is available under the MIT license at our project webpage (https://iba.yaplab.io).

For manuscripts utilizing custom algorithms or software that are central to the research but not yet described in published literature, software must be made available to editors and reviewers. We strongly encourage code deposition in a community repository (e.g. GitHub). See the Nature Research guidelines for submitting code & software for further information.

Data

Policy information about availability of data

All manuscripts must include a data availability statement. This statement should provide the following information, where applicable:
- Accession codes, unique identifiers, or web links for publicly available datasets
- A list of figures that have associated raw data
- A description of any restrictions on data availability

The infant brain atlases are available at Zenodo (https://doi.org/10.5281/zenodo.7044932) under the Creative Commons Attribution Non Commercial Share Alike 4.0 International license.

The MRI data used in this work can be obtained from the National Institute of Mental Health Data Archive (NDA, https://nda.nih.gov/) or by contacting the investigative team. Source data for quantitative results in the main Figures 4, 5, and 6, Extended Data Figures 8 and 10, and Supplementary Figures 5, 7, 8, 10, 12, 13, and 17 are provided as Excel spreadsheets with this paper.

Field-specific reporting

Please select the one below that is the best fit for your research. If you are not sure, read the appropriate sections before making your selection.

☒ Life sciences ☐ Behavioural & social sciences ☐ Ecological, evolutionary & environmental sciences

For a reference copy of the document with all sections, see nature.com/documents/nr-reporting-summary-flat.pdf

Life sciences study design

All studies must disclose on these points even when the disclosure is negative.

Sample size	Longitudinal T1w and T2w MRI scans were acquired for 37 subjects (20 females; 17 males) enrolled as part of the UNC/UMN Baby Connectome Project (BCP). The subjects enrolled in the BCP study were divided into six cohorts (A1, A2, A3, B1, B2, B3), and each cohort's first visit was scheduled at 2 weeks, 1, 2, 9, 10, and 11 months, respectively. The subjects in A1, A2, and A3 were scheduled to be scanned every three months in the first year and then at 24 months; whereas, the subjects in B1, B2, and B3 were scanned every three months for the first two years. The total number of scans for each subject is different since all subjects cannot be scanned at all expected time points. A total of 108 scans for each imaging modality were used in the current work.<br><br>Empirical results indicate that brain atlas variability decreases with sample size and reaches a subvoxel level with 30 samples (Supplementary Note 4). Thus, 108 longitudinal MRI scans from 37 subjects are sufficient for the purpose of this work.
Data exclusions	Data affected by artifacts, such as those caused by motion, were not included in our dataset.
Replication	We showed and discussed at length that our atlases reproduce findings observed in previous studies about brain development.
Randomization	Randomization is not applicable because this study does not involve comparison between groups.
Blinding	Blinding is not applicable because this study does not involve comparison between groups.

Reporting for specific materials, systems and methods

We require information from authors about some types of materials, experimental systems and methods used in many studies. Here, indicate whether each material, system or method listed is relevant to your study. If you are not sure if a list item applies to your research, read the appropriate section before selecting a response.

Materials & experimental systems

n/a	Involved in the study	
☒	☐	Antibodies
☒	☐	Eukaryotic cell lines
☒	☐	Palaeontology and archaeology
☒	☐	Animals and other organisms
☐	☒	Human research participants
☒	☐	Clinical data
☒	☐	Dual use research of concern

Methods

n/a	Involved in the study	
☒	☐	ChIP-seq
☒	☐	Flow cytometry
☐	☒	MRI-based neuroimaging

Human research participants

Policy information about studies involving human research participants

Population characteristics	Typically developing infants (51% female, 49% male), aged between birth and 5 years, were enrolled as part of the UNC/UMN Baby Connectome Project (BCP).
Recruitment	Participants were recruited from existing registries at UNC and UMN based on state-wide birth records as well as from broader community resources (e.g., community centers and targeted day-care centers) to ensure the sample approximates the racial/ethnic and socio-economic diversity of the US census.
Ethics oversight	The study protocols were approved by the Institutional Review Board of the School of Medicine of the University of North Carolina at Chapel Hill (UNC-CH), NC, USA.

Note that full information on the approval of the study protocol must also be provided in the manuscript.

Magnetic resonance imaging

Experimental design

Design type	Not applicable; only structural MRI, involving T1-weighted and T2-weighted images, was used.
Design specifications	Not applicable; only structural MRI, T1-weighted and T2-weighted images, was used.
Behavioral performance measures	Not applicable; only structural MRI, T1-weighted and T2-weighted images, was used.

Acquisition

Imaging type(s)	Structural T1-weighted and T2-weighted imaging.
Field strength	3T
Sequence & imaging parameters	MPRAGE, SPACE, FOV: 256mm x 256mm, slice thickness: 0.8mm.
Area of acquisition	Whole brain.

Diffusion MRI ☐ Used ☒ Not used

Preprocessing

Preprocessing software	iBEAT v2.0 (https://ibeat.wildapricot.org) for tissue segmentation maps and cortical surfaces.
Normalization	Data were normalized using rigid transform.
Normalization template	In-house brain template.
Noise and artifact removal	Bias-field correction.
Volume censoring	Not applicable. Only structural MRI, involving T1-weighted and T2-weighted images, was used. Images were quality-controlled before they were used.

Statistical modeling & inference

Model type and settings	Generalized additive mixture model (GAMM) and generalized additive model (GAM) with cubic regression, as described in the Methods section of the manuscript.
Effect(s) tested	Not applicable.

Specify type of analysis: ☒ Whole brain ☐ ROI-based ☐ Both

Statistic type for inference (See Eklund et al. 2016)	Models were fitted at the vertex level with goodness of fit determined via adjusted R^2.
Correction	Not applicable. Multiple comparisons not involved.

Models & analysis

n/a	Involved in the study
☒	☐ Functional and/or effective connectivity
☒	☐ Graph analysis
☒	☐ Multivariate modeling or predictive analysis
